# Supplementary material for: Haplotype-resolved assembly of a tetraploid potato genome using long reads and low-depth offspring data
Source: Genome Biol. 2024 Jan 19;25:26. doi: 10.1186/s13059-023-03160-z (PMC10797741; doi:10.1186/s13059-023-03160-z)
Supplement: Supplementary file 1 — Additional file 1: Fig. S1. Bandage visualization of the hifiasm raw unitig graph. Fig. S2. Dosage distribution of unitigs. Fig S3. Joint coverage of ONT reads and HiFi reads, mapped to the hifiasm assembly graph of Altus. Fig S4. UpSet plot of the various node sets used throughout this study. Fig S5. Genome size estimation using GenomeScope. Fig S6. Mapping of the contigs from all four haplotype clusters of chromosome 2 to the DMv6.1 reference. Fig S7. Haplotype-resolved version of (main) Fig. 4a. Fig. S8. Analysis of assembly errors in the hifiasm graph between chromosomes 10 and 12 of DMv6.1. Fig. S9. Mapping of the clusters to the Solyntus v1.1 reference sequence. Fig. S10. Analysis of assembly inserts between chromosomes 1 and 8 in the Solyntus v1.1 reference sequence. Figs. S11-S16. Synteny analysis between assembled haplotypes and DMv6.1. Figs. S17-S28. Synteny analysis between all four haplotypes per chromosome. Fig. S29. Pore-C data analysis for phasing validation: ROC curves for four different distance cutoffs. [file 13059_2023_3160_MOESM1_ESM.pdf]

SUPPLEMENTARY MATERIAL:

Haplotype-resolved assembly of a tetraploid potato genome using long reads and low-depth offspring data

Rebecca Serra Mari[1,9], Sven Schrinner[2], Richard Finkers[3,4], Freya Maria Rosemarie Ziegler[5,6,7,8], Paul Arens[4], Maximilian H.-W. Schmidt[5,6], Björn Usadel[5,6,7,8,**], Gunnar W. Klau[2,5,**], Tobias Marschall[1,9,**]

[1] Institute for Medical Biometry and Bioinformatics, Medical Faculty and University Hospital Düsseldorf, Heinrich Heine University Düsseldorf, Germany

[2] Algorithmic Bioinformatics, Faculty of Mathematics and Natural Sciences, Heinrich Heine University Düsseldorf, Germany

[3] Gennovation B.V., Agro Business Park 10, 6708 PW, Wageningen, The Netherlands

[4] Plant Breeding, Wageningen University & Research, The Netherlands

[5] Cluster of Excellence on Plant Sciences (CEPLAS), Heinrich Heine University Düsseldorf, Germany

[6] Forschungszentrum Jülich, Institute of Bio and Geosciences, Bioinformatics (IBG-4), Germany

[7] Bioeconomy Science Center, c/o Forschungszentrum Jülich, Germany

[8] Biological Data Science, Faculty of Mathematics and Natural Sciences, Heinrich Heine University Düsseldorf, Germany

[9] Center for Digital Medicine, Heinrich Heine University Düsseldorf, Germany

** Correspondence to tobias.marschall@hhu.de, gunnar.klau@hhu.de, b.usadel@fz-juelich.de

Initial assembly

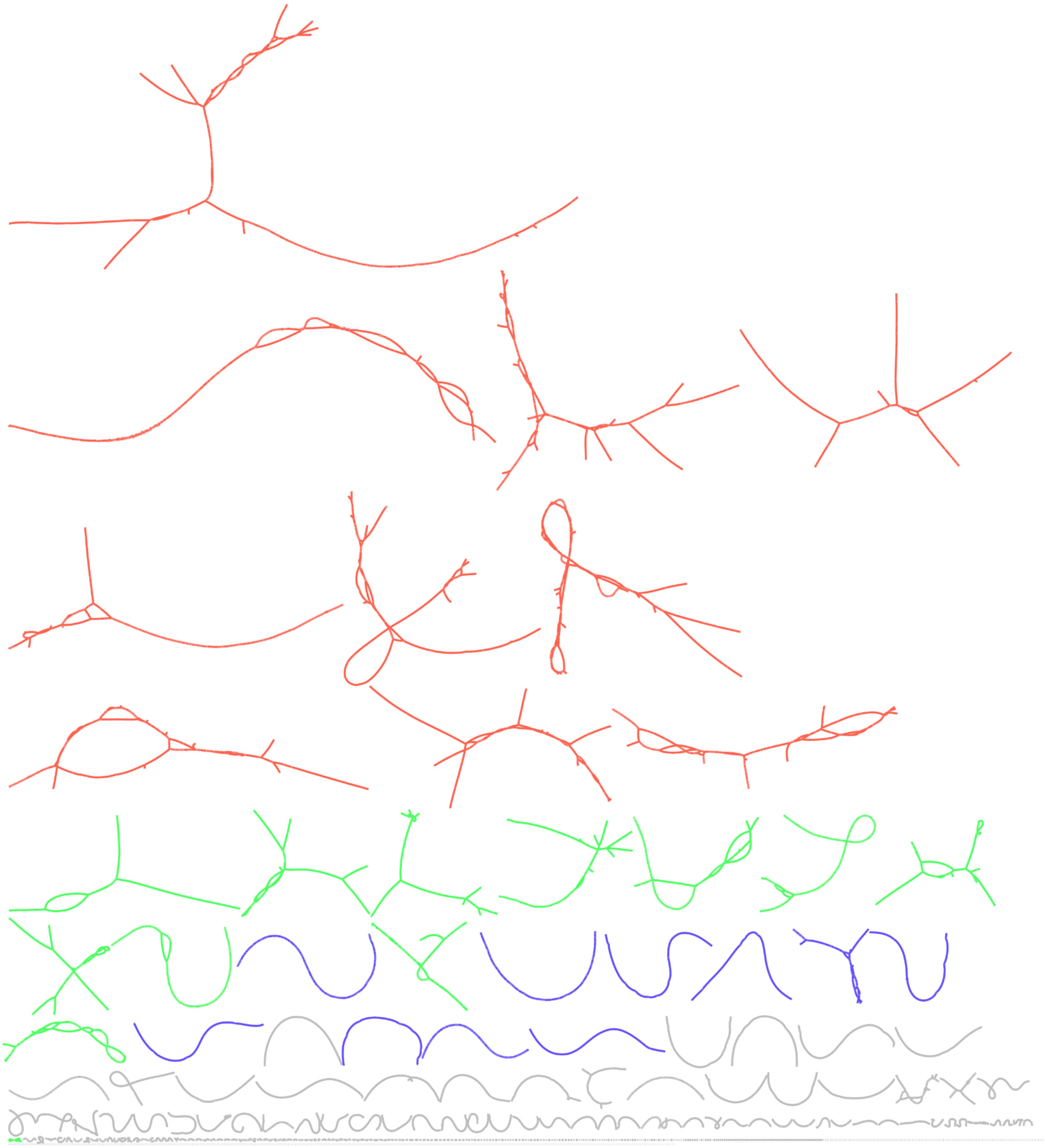

Fig. S1: Bandage visualisation of the hifiasm raw unitig graph.

The different size categories are indicated by the colouring: red represents the largest components (91–190 Mb), green the second largest (45–66 Mb), and blue the third largest (20–32 Mb).

Dosage analysis

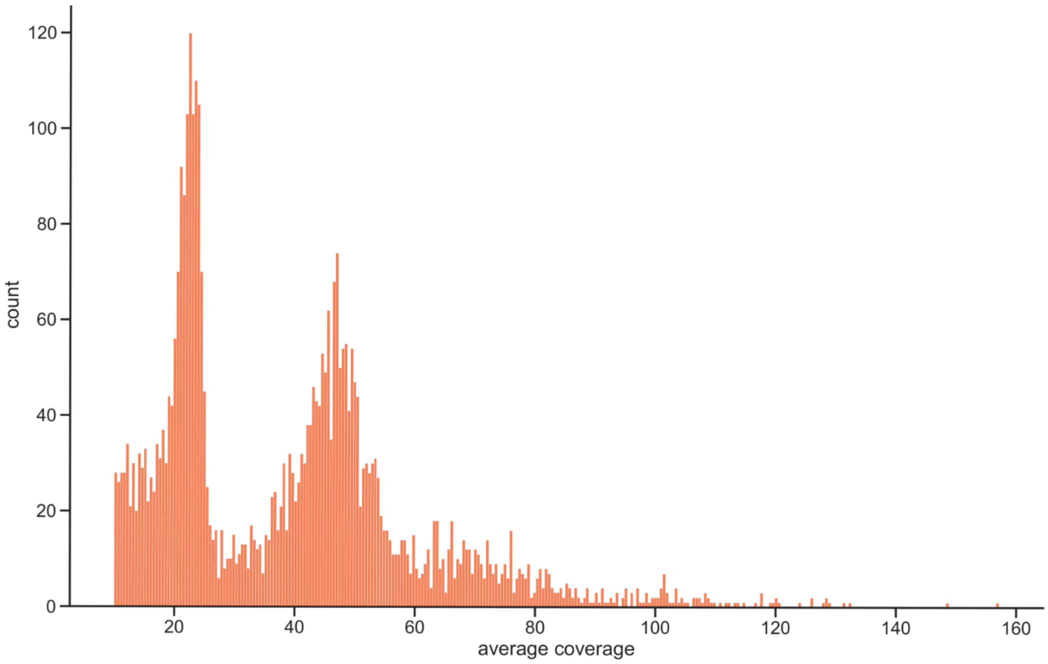

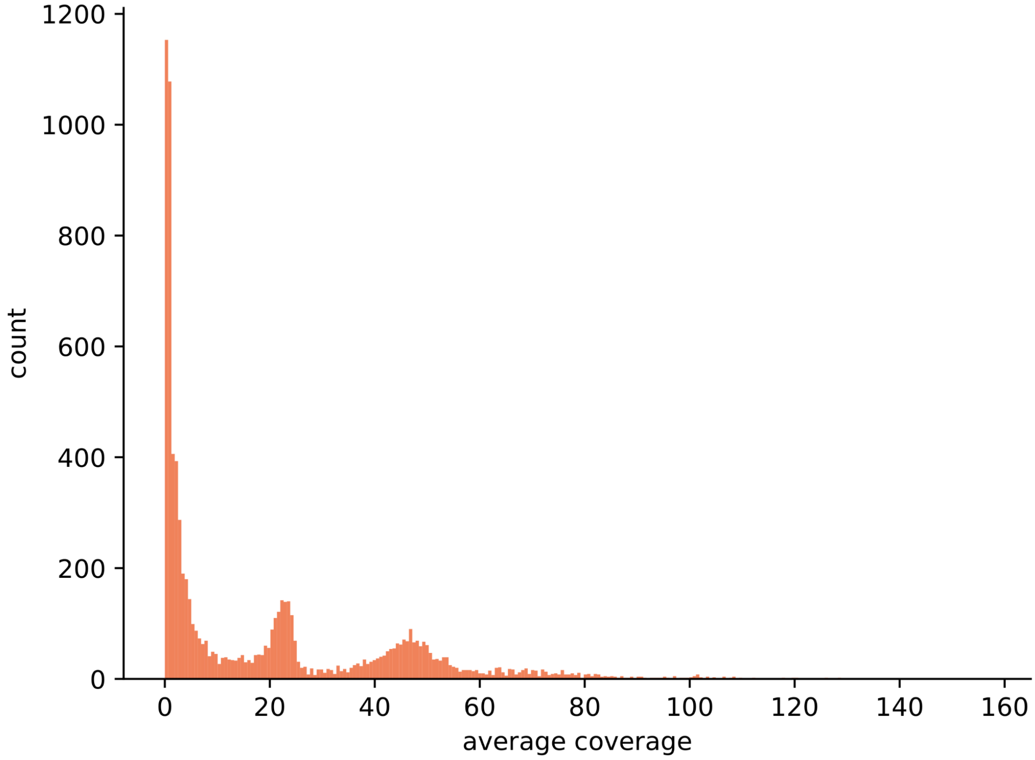

Fig. S2: Dosage distribution of unitigs. Above: Contigs with coverage < 10 are filtered out for better visualisation. Below: All contigs are shown.

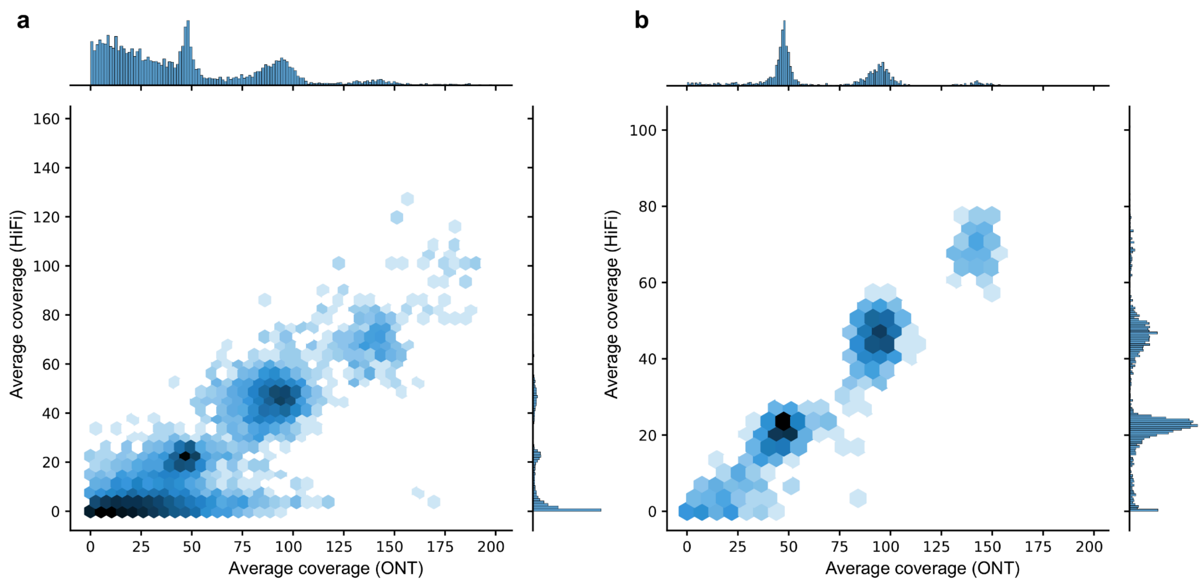

Fig S3: Joint coverage of ONT reads and HiFi reads, mapped to the hifiasm assembly graph of Altus.
a: All nodes without any filtering applied, b: Filtered to only show coverage of nodes with a unique sequence length of at least 100kb.

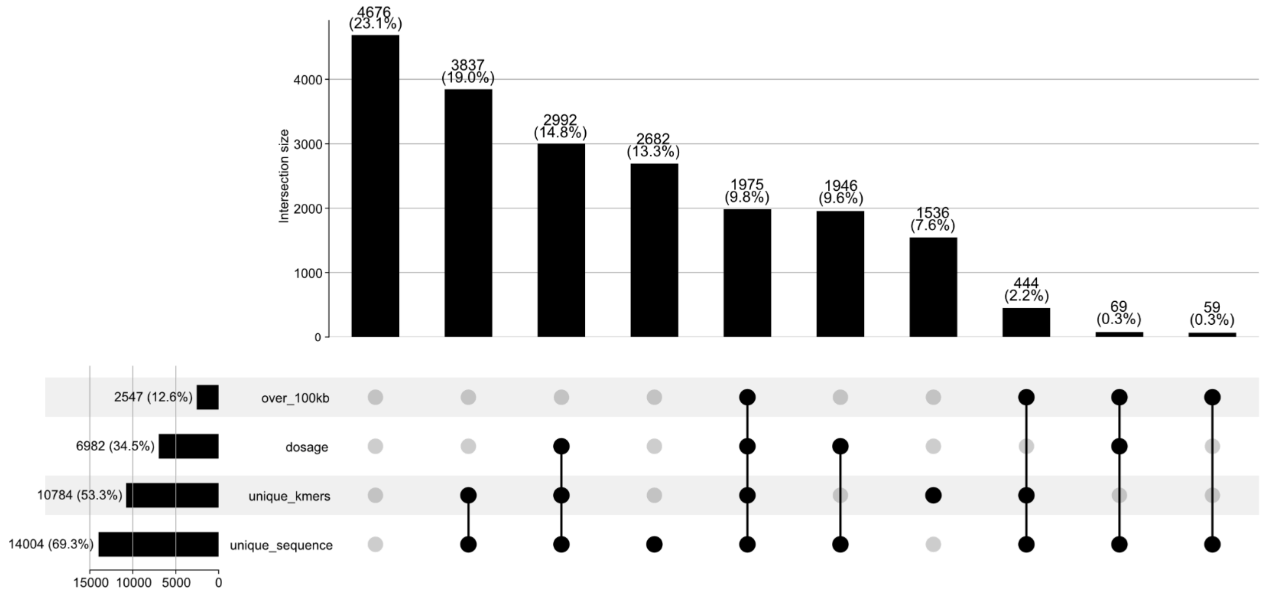

Fig S4: UpSet plot of the various node sets used throughout this study. 'Node' refers to the unitigs from the initial assembly graph. 'Over_100kb': nodes with a length of at least 100 kb. 'Dosage': Nodes for which we obtained dosage information, i.e. the dosage estimate of HiFi and ONT reads coincides. 'Unique_kmers': Nodes that contain >0 unique k-mers. 'Unique_sequences': Nodes for which the unique – i.e. the non-overlapping – sequence is longer than 0bp.

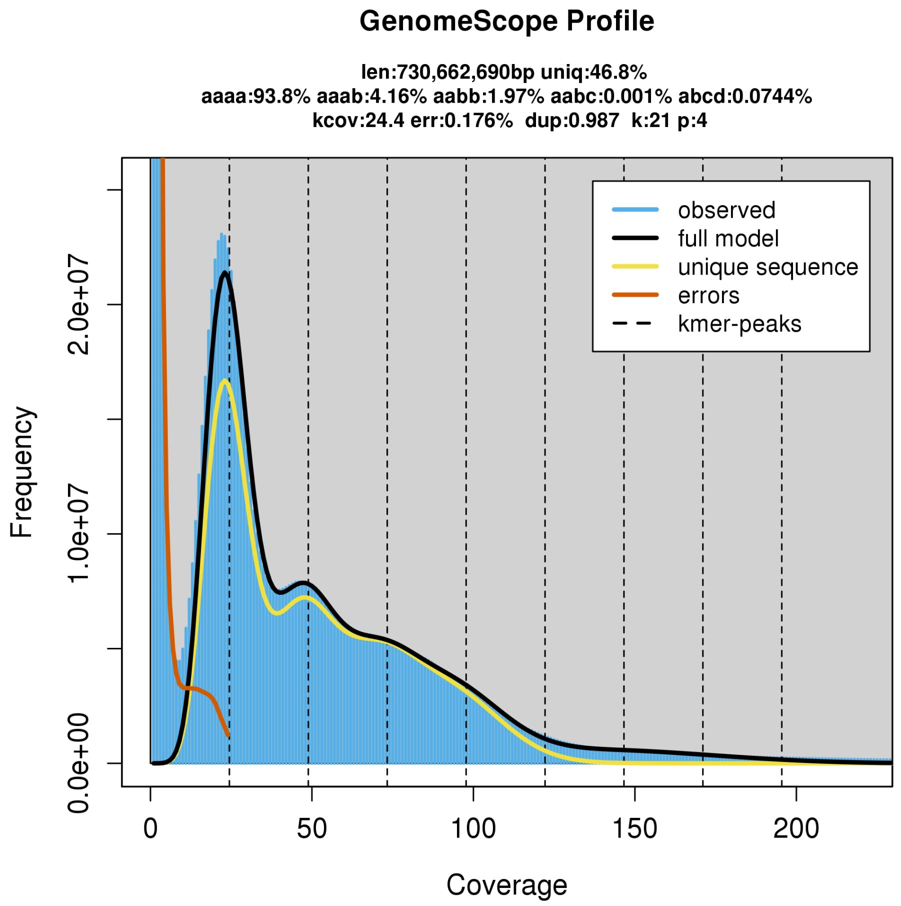

Fig S5: Genome size estimation using GenomeScope.

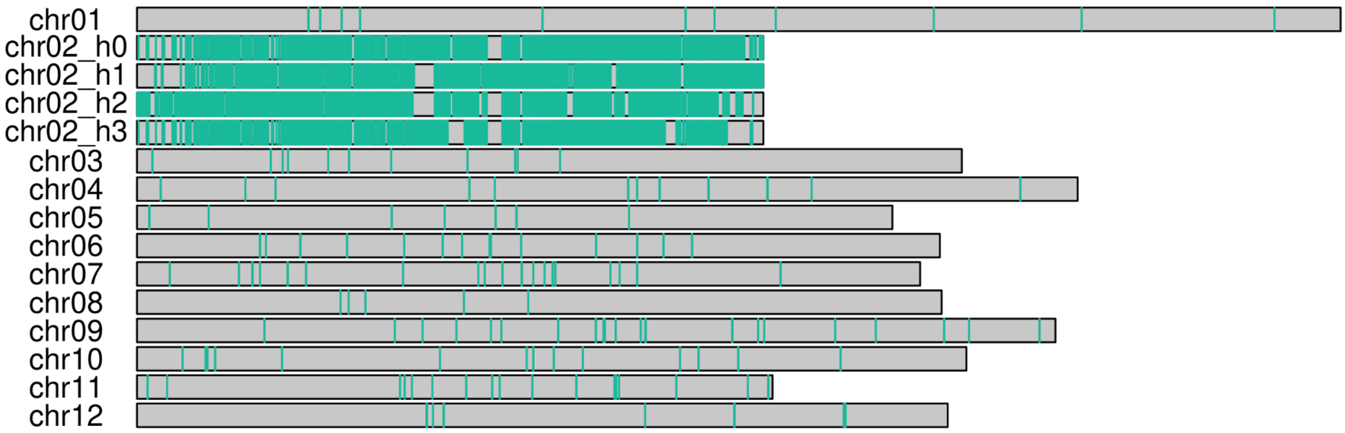

Fig S6: Mapping of the contigs from all four haplotype clusters of chromosome 2 to the DMv6.1 reference.

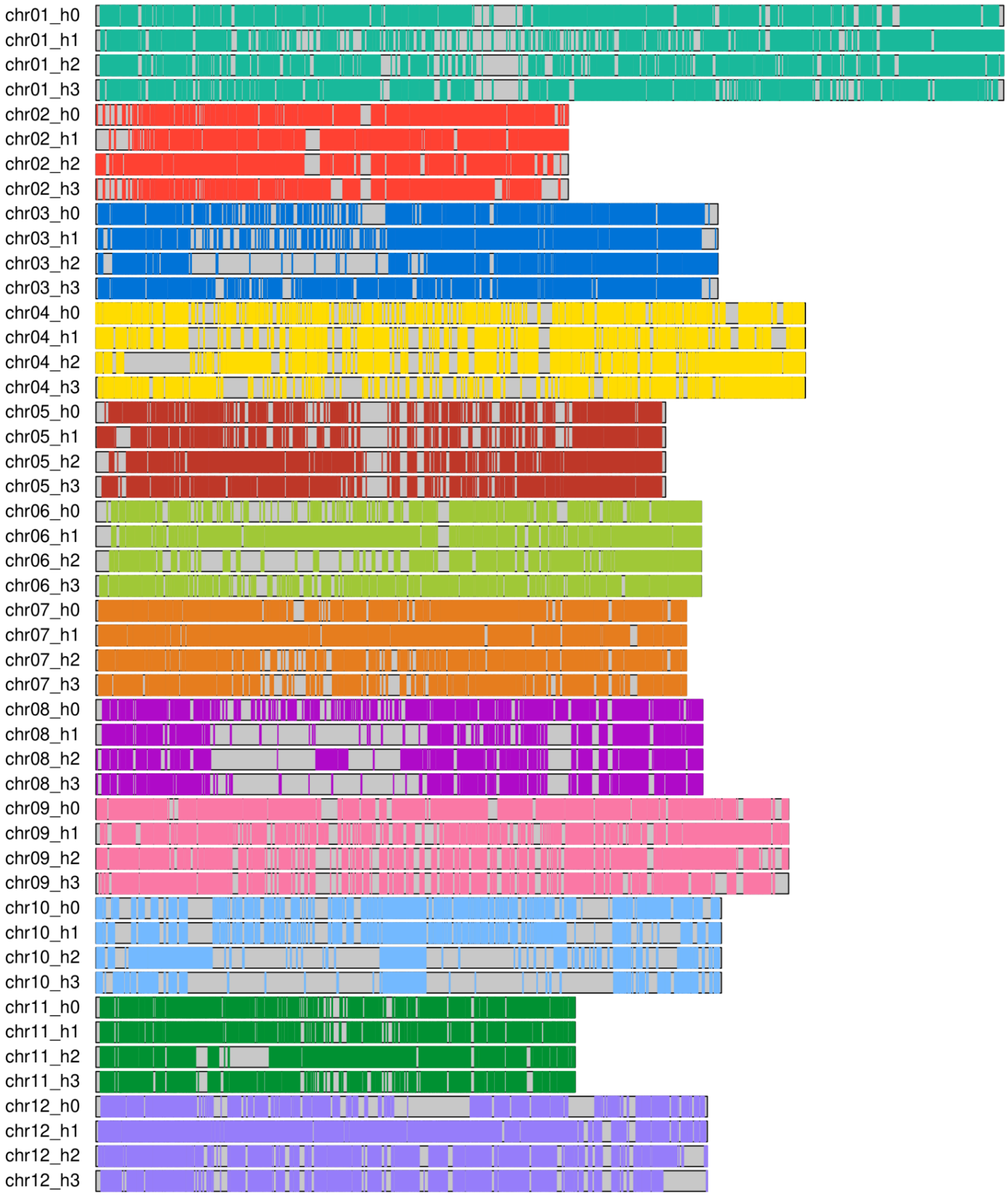

Fig S7: Haplotype-resolved version of (main) Fig. 4a. All contigs from the 48 haplotype clusters have been mapped to the reference DMv6.1.

Comparison of earlier reference assemblies to reveal structural differences

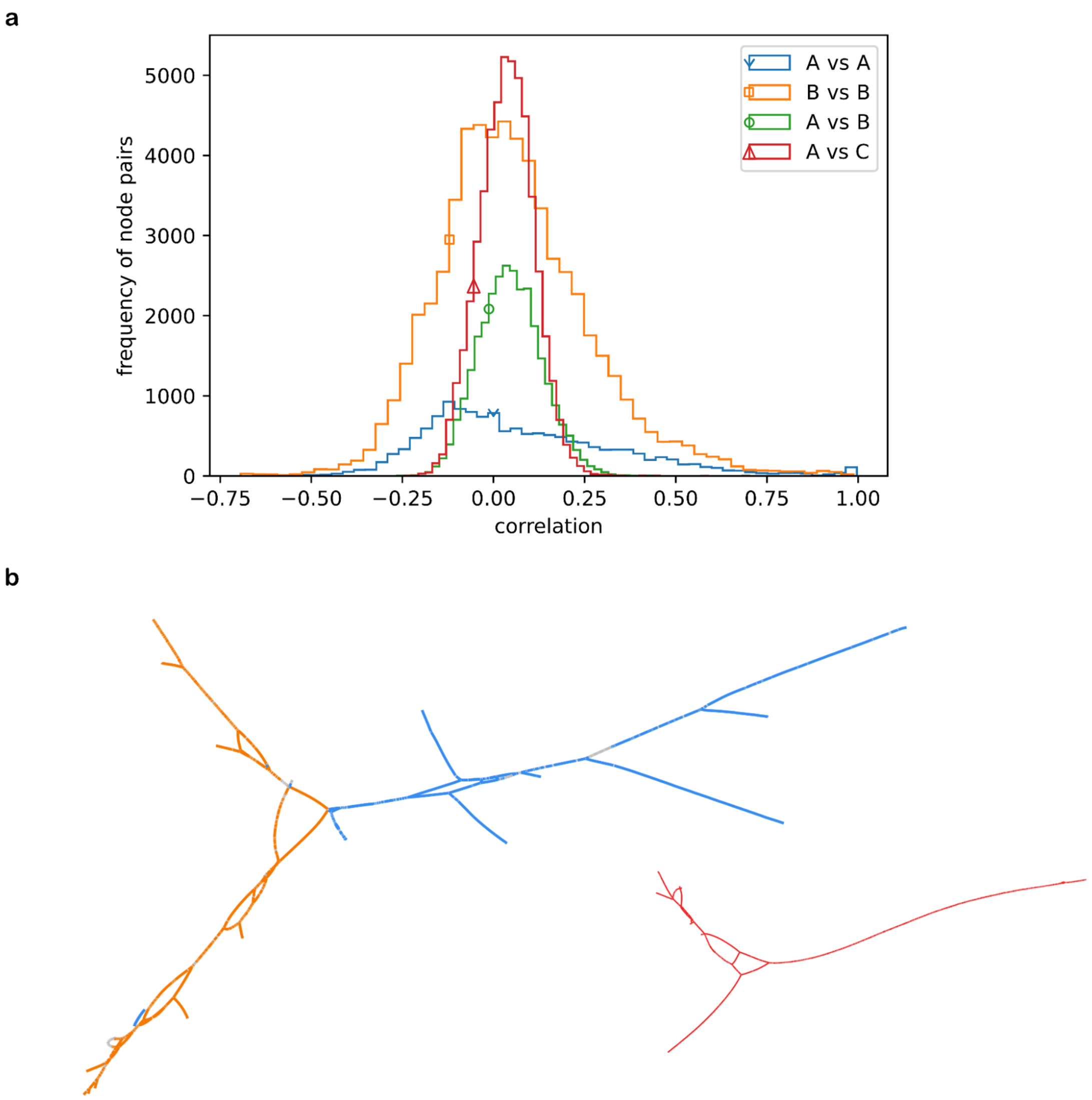

Fig. S8: Analysis of assembly errors in the hifiasm graph between chromosomes 10 and 12 of DMv6.1.

a. Distribution of the pairwise correlation of nodes contained within set A (blue), within set B (orange), between sets A and B (green), and between A and an arbitrarily chosen different component C (red).
b. Left: The component of the assembly graph that contains node sets A (contigs that map to chromosome 12 in the DMv6.1 sequence, colored blue) and B (contigs that map to chromosome 10 in the DMv6.1 sequence, colored orange). Right: The component used for comparison, labelled C (contigs are colored in red).

a

b

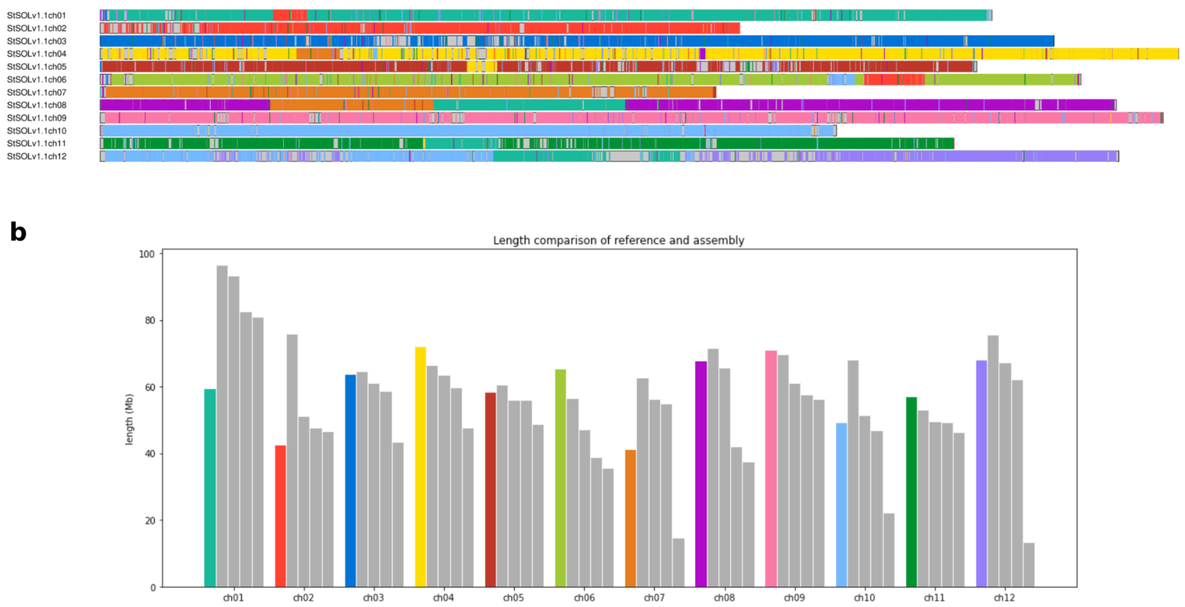

Fig. S9: Mapping of the clusters to the Solyntus v1.1 reference sequence.

a

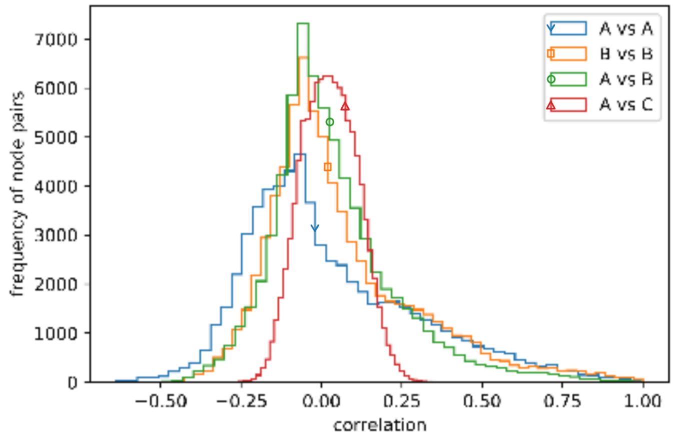

b

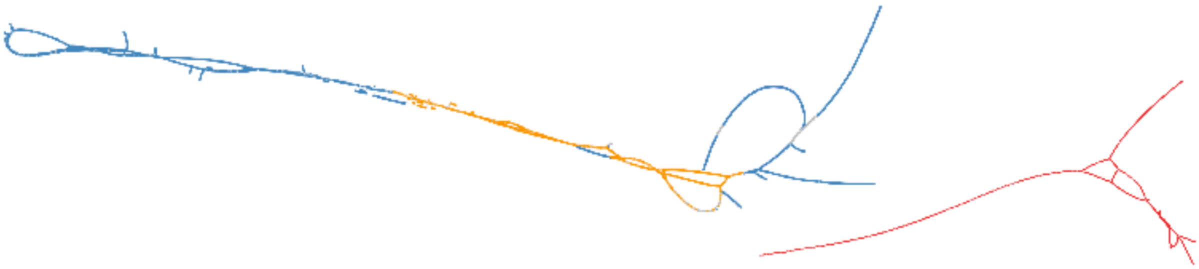

Fig. S10: Analysis of assembly inserts between chromosomes 1 and 8 in the Solyntus v1.1 reference sequence.

a. Distribution of the pairwise correlation of nodes contained within set A (blue), within set B (orange), between sets A and B (green), and between A and an arbitrarily chosen different component C (red).
b. Left: The component of the assembly graph that contains node sets A (contigs that map to chromosome 1, colored blue) and B (contigs that map to chromosome 8, colored orange). Right: The component used for comparison, labelled C (contigs are colored in red).

Synteny analysis

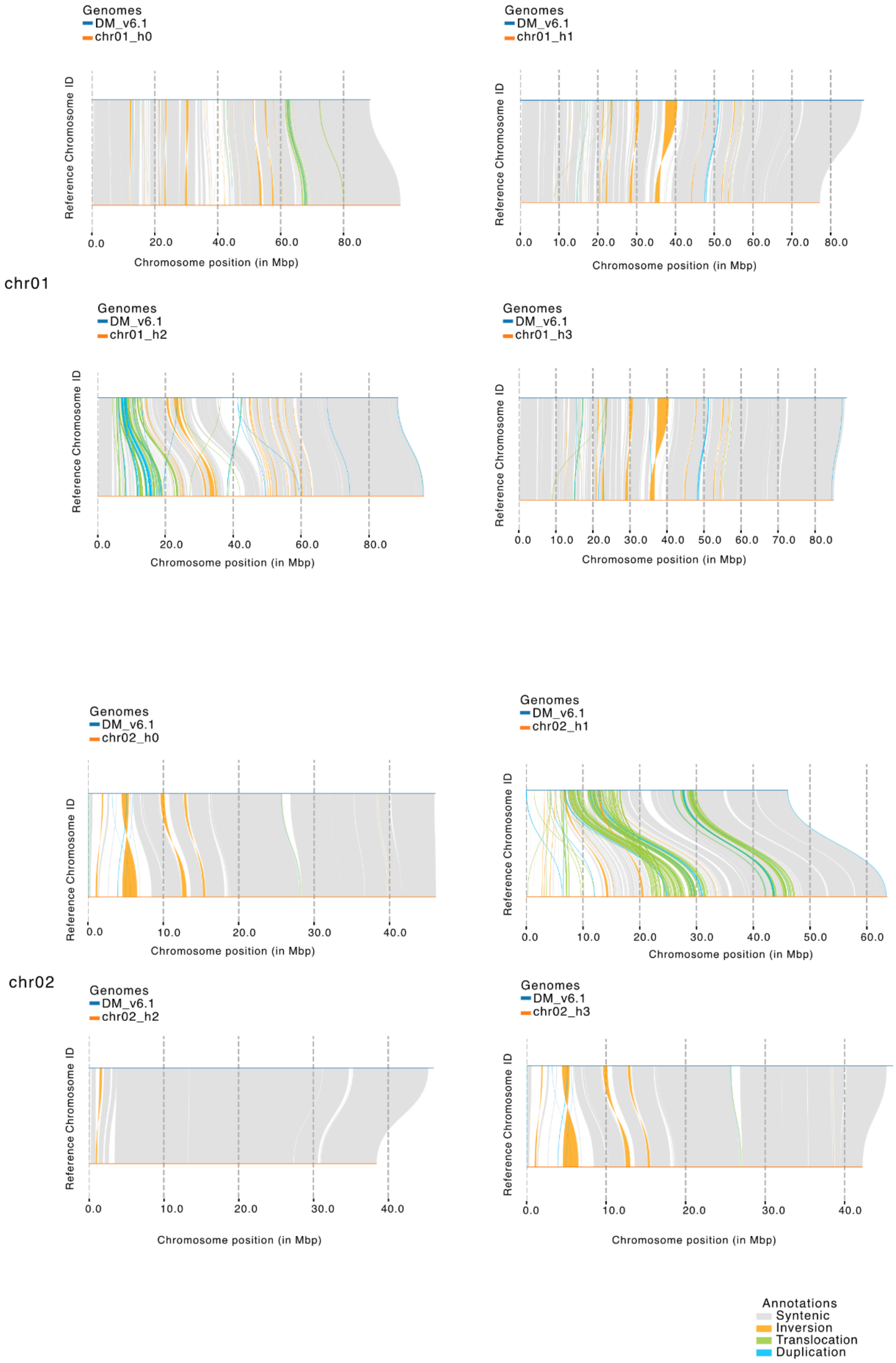

Fig. S11: Synteny analysis. Comparison between all haplotypes of chr01 (above) and chr02 (below) and the reference sequence DMv6.1.

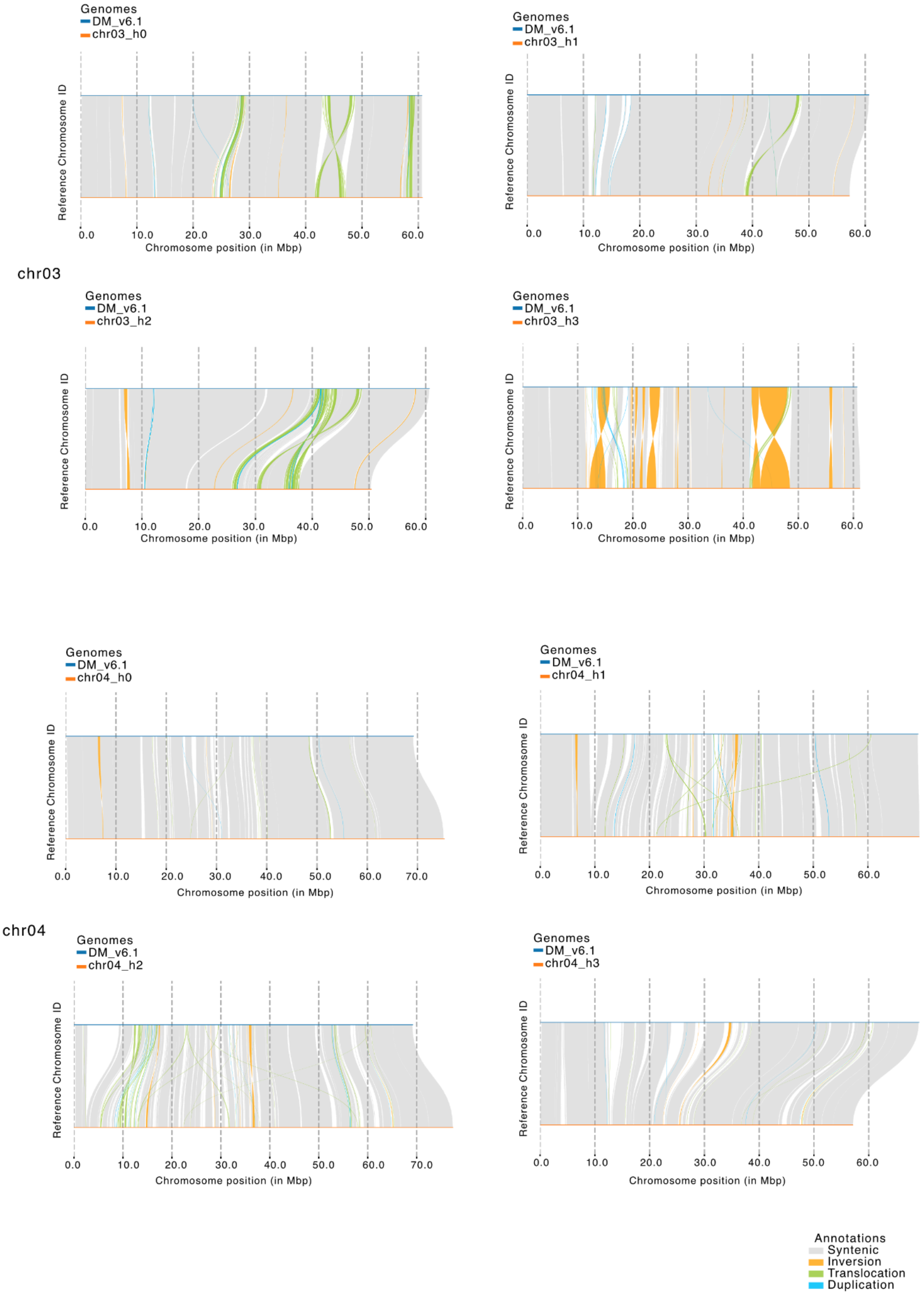

Fig. S12: Synteny analysis. Comparison between all haplotypes of chr03 (above) and chr04 (below) and the reference sequence DMv6.1.

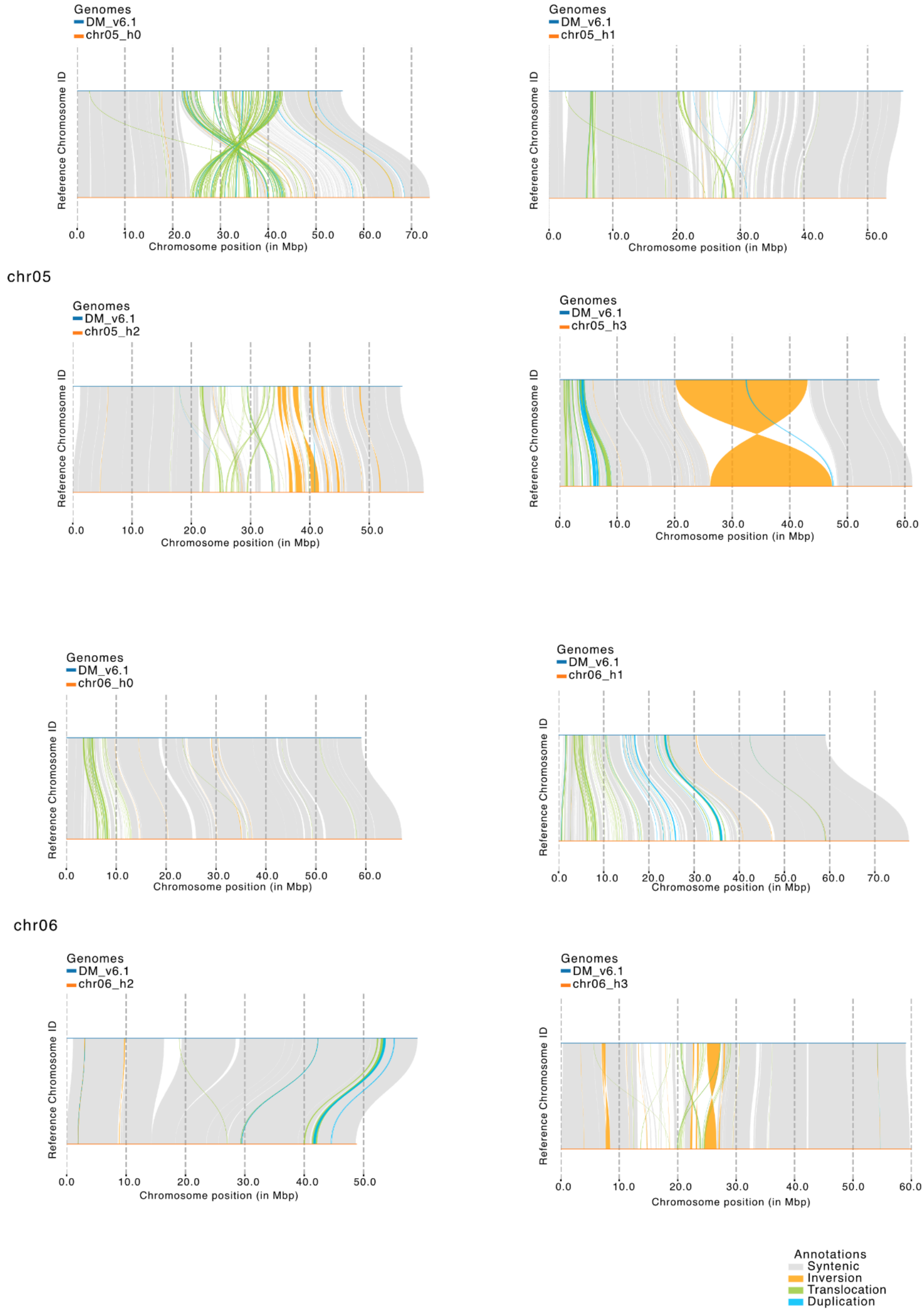

Fig. S13: Synteny analysis. Comparison between all haplotypes of chr05 (above) and chr06 (below) and the reference sequence DMv6.1.

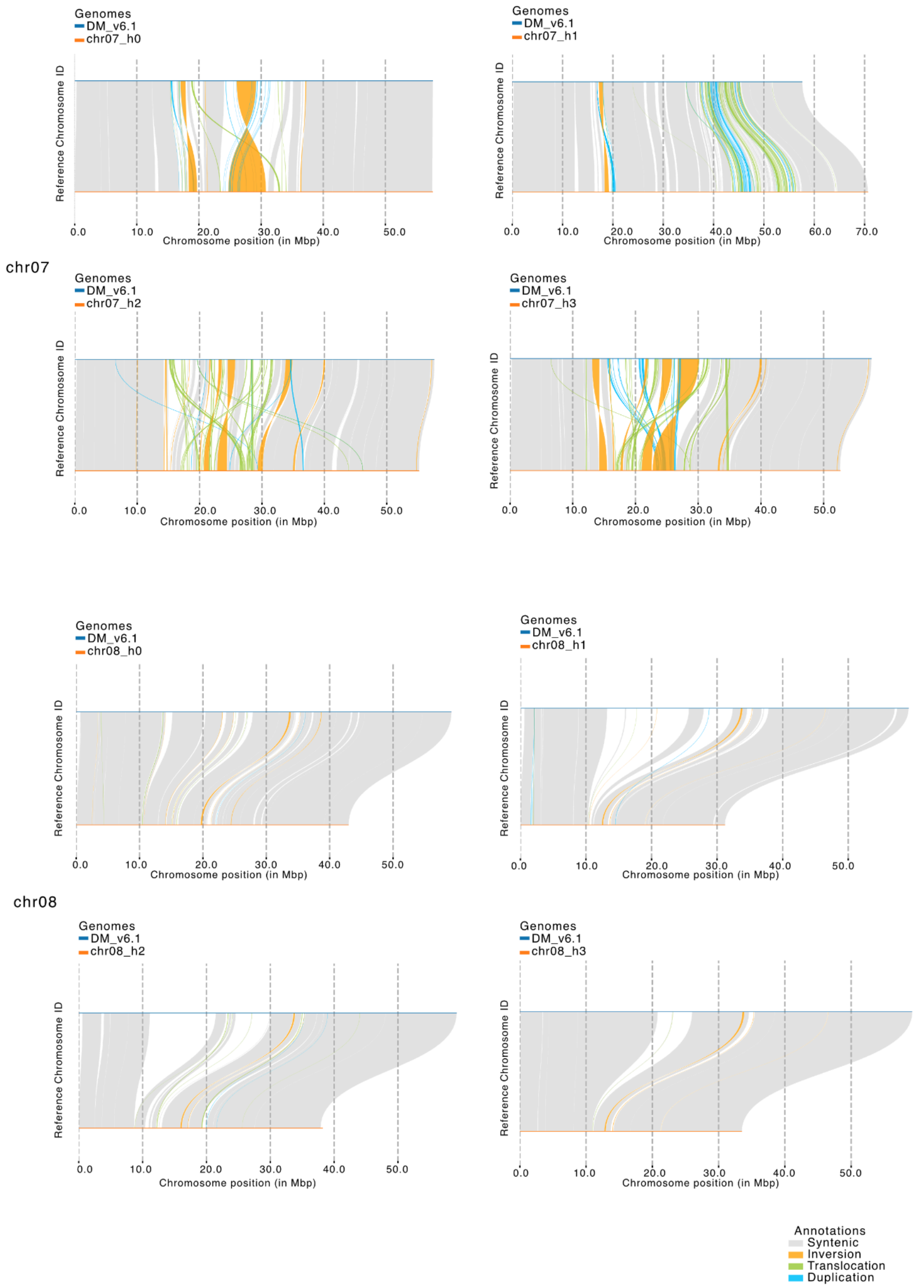

Fig. S14: Synteny analysis. Comparison between all haplotypes of chr07 (above) and chr08 (below) and the reference sequence DMv6.1.

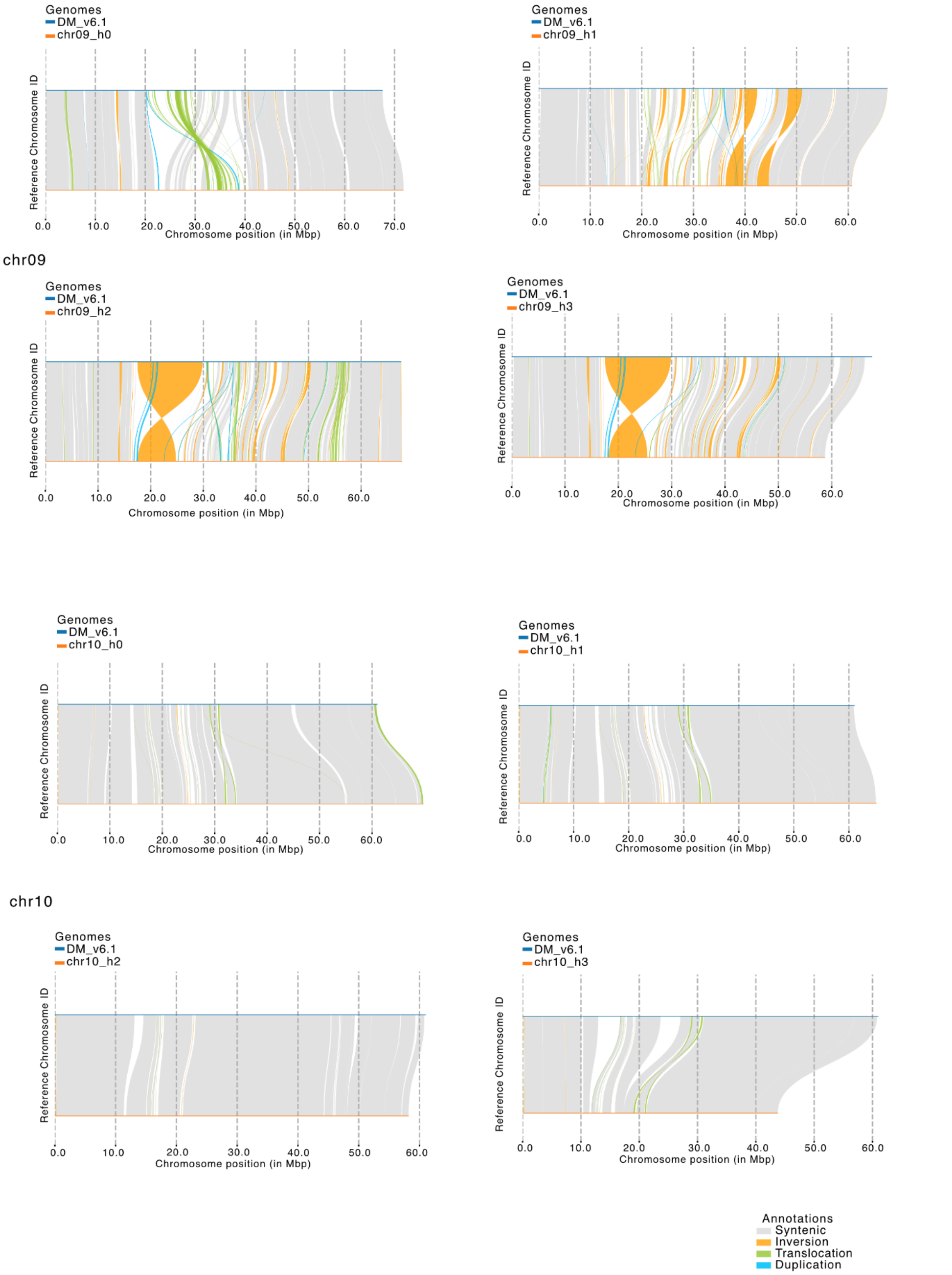

Fig. S15: Synteny analysis. Comparison between all haplotypes of chr09 (above) and chr10 (below) and the reference sequence DMv6.1.

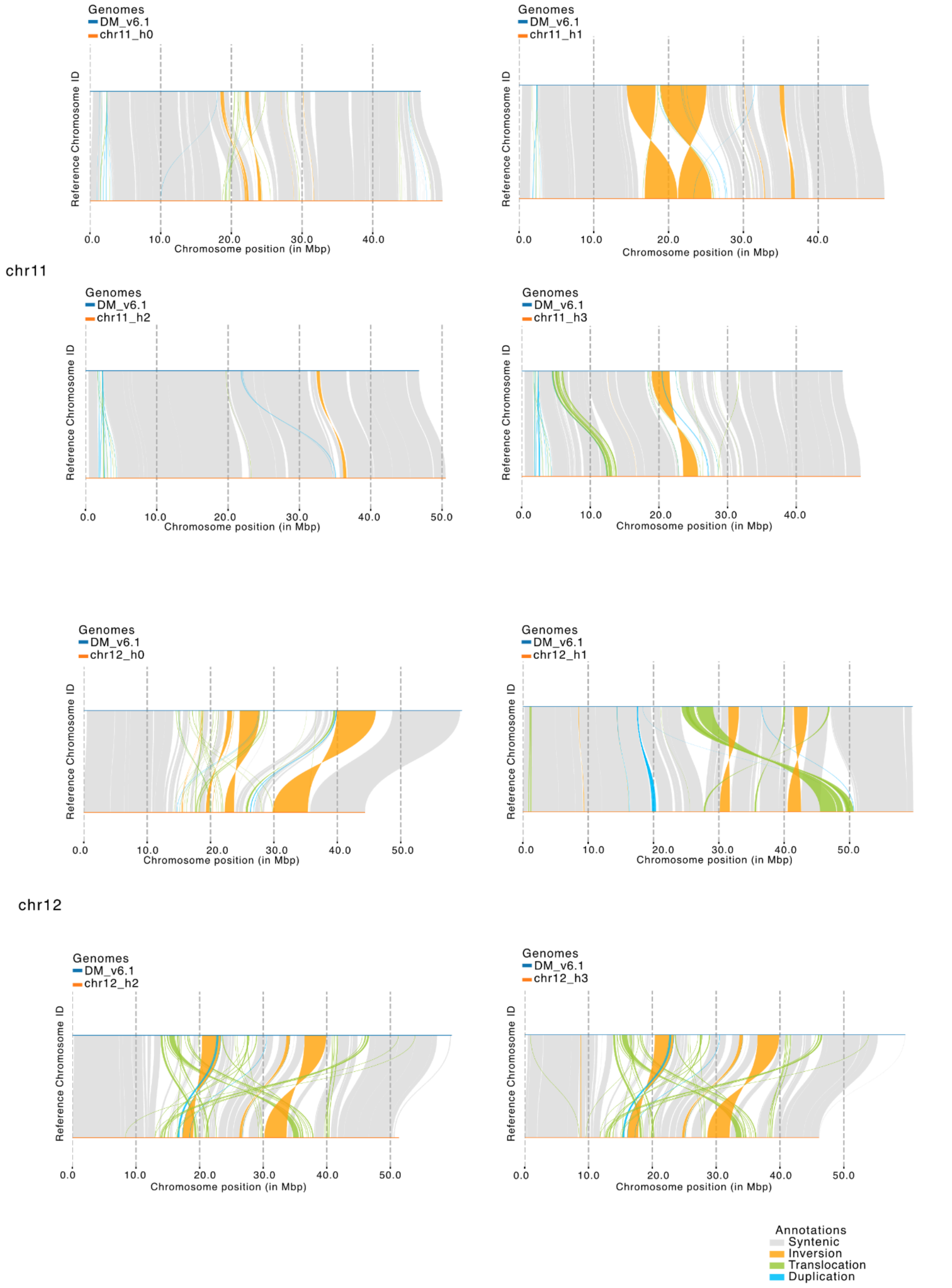

Fig. S16: Synteny analysis. Comparison between all haplotypes of chr11 (above) and chr12 (below) and the reference sequence DMv6.1.

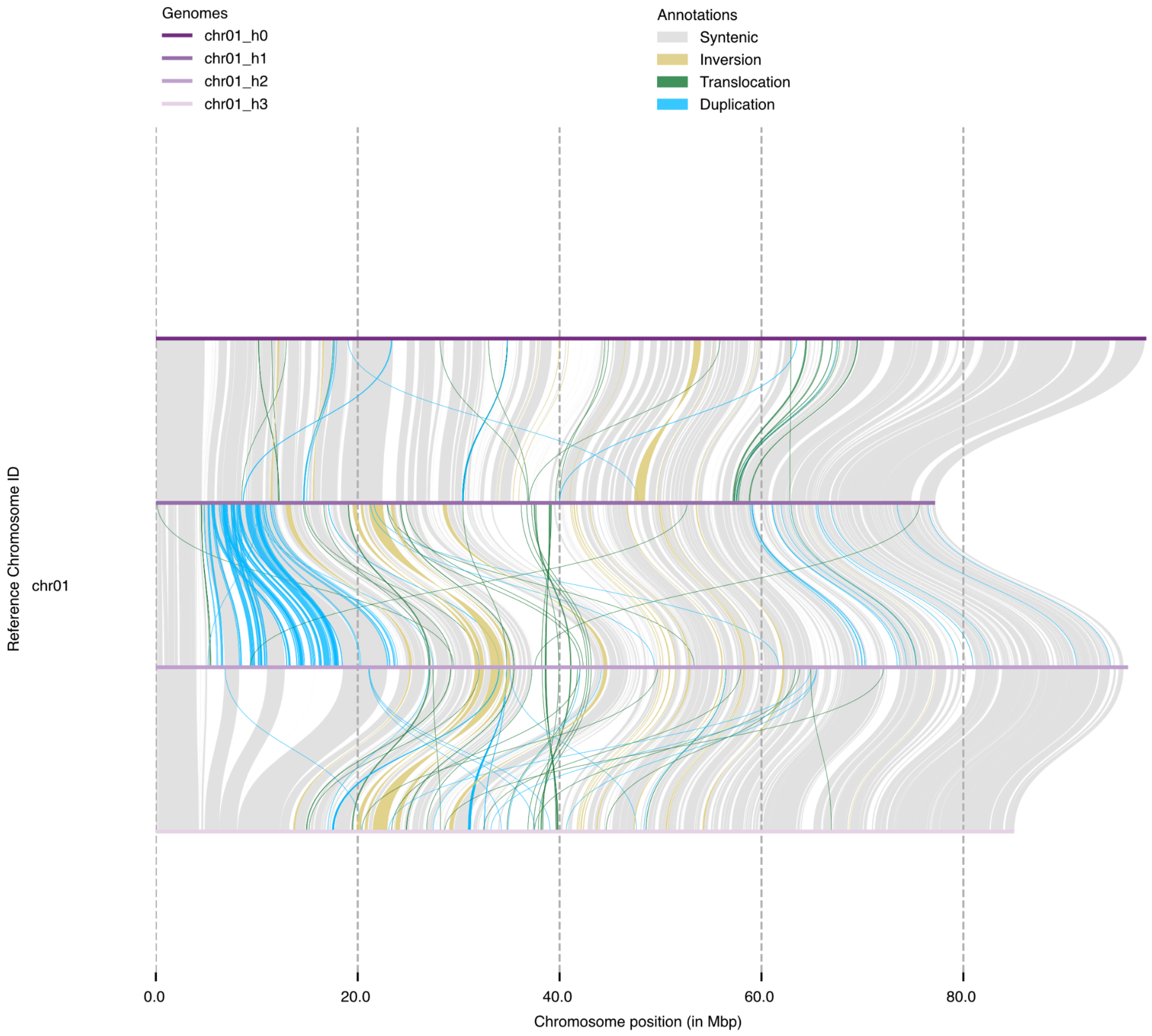

Fig. S17: Synteny analysis of chr01.

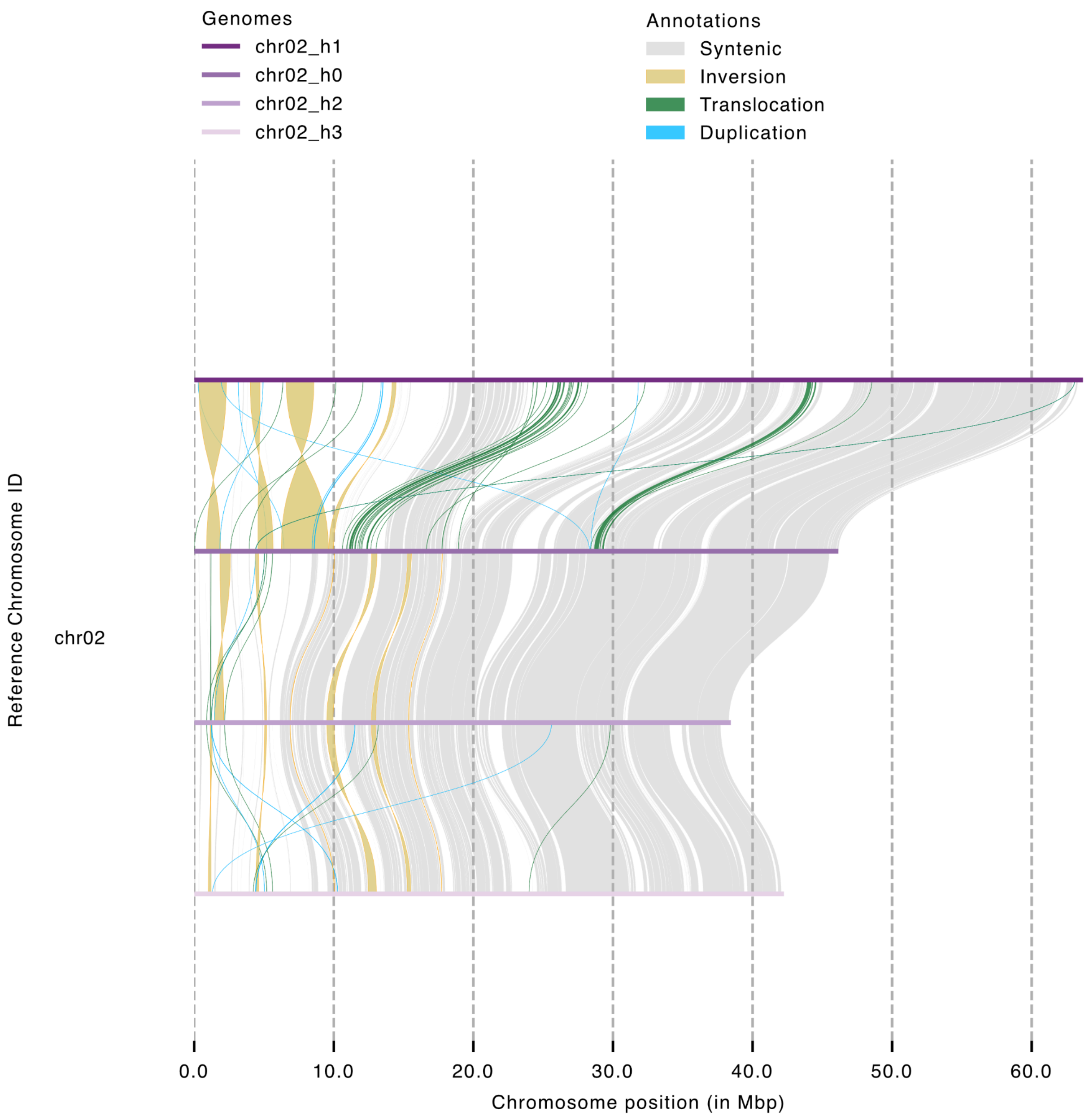

Fig. S18: Synteny analysis of chr02.

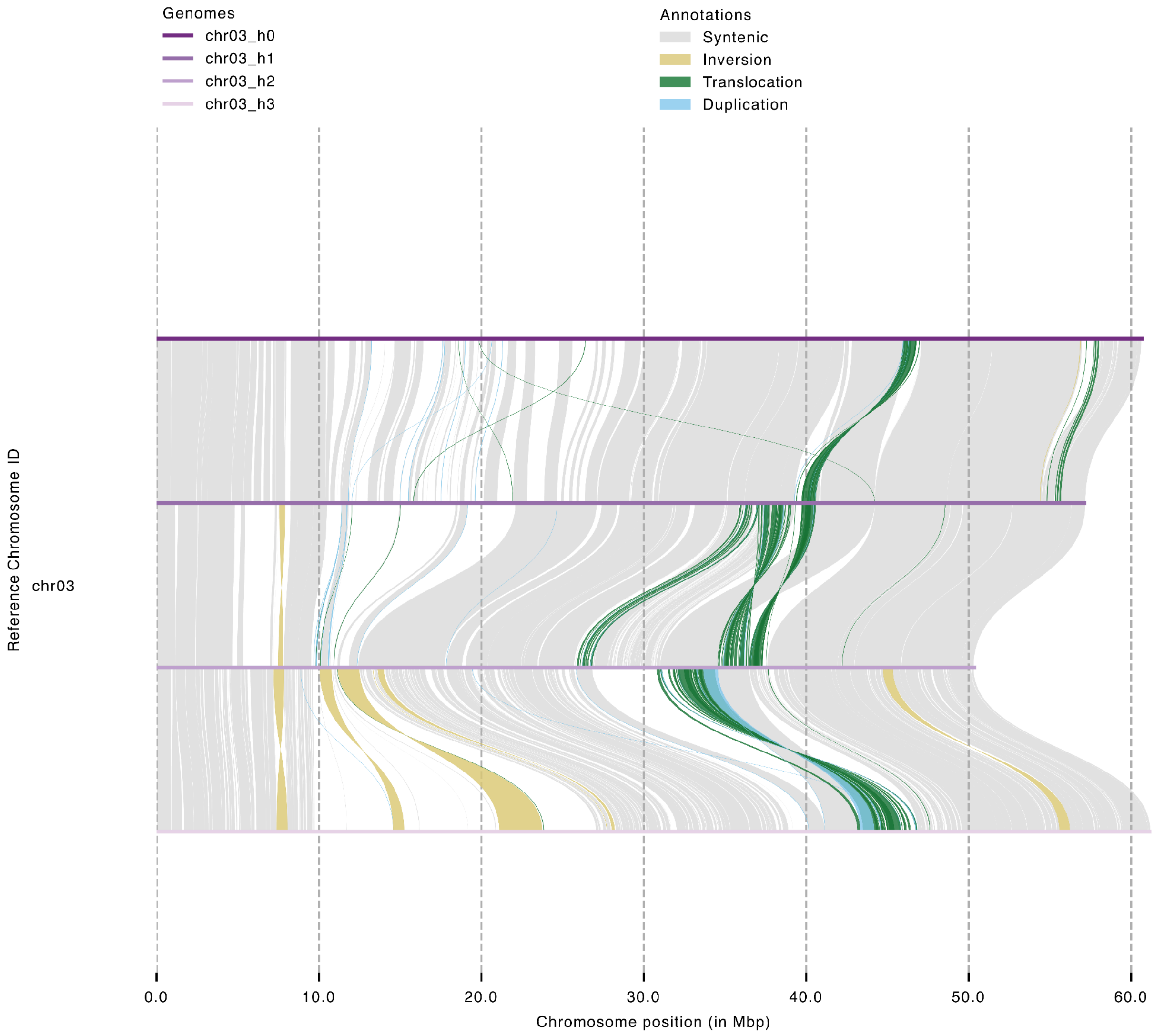

Fig. S19: Synteny analysis of chr03.

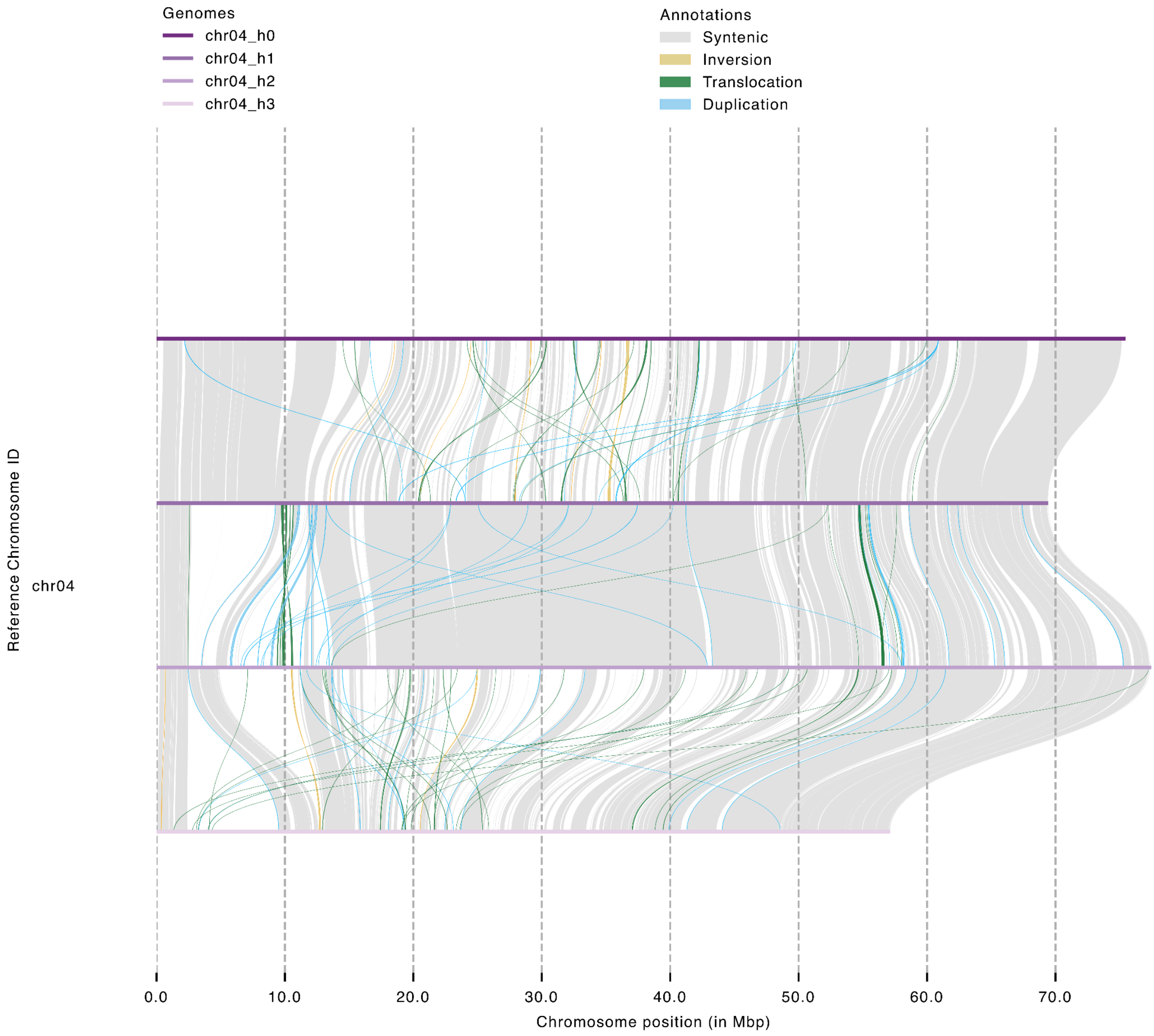

Fig. S20: Synteny analysis of chr04.

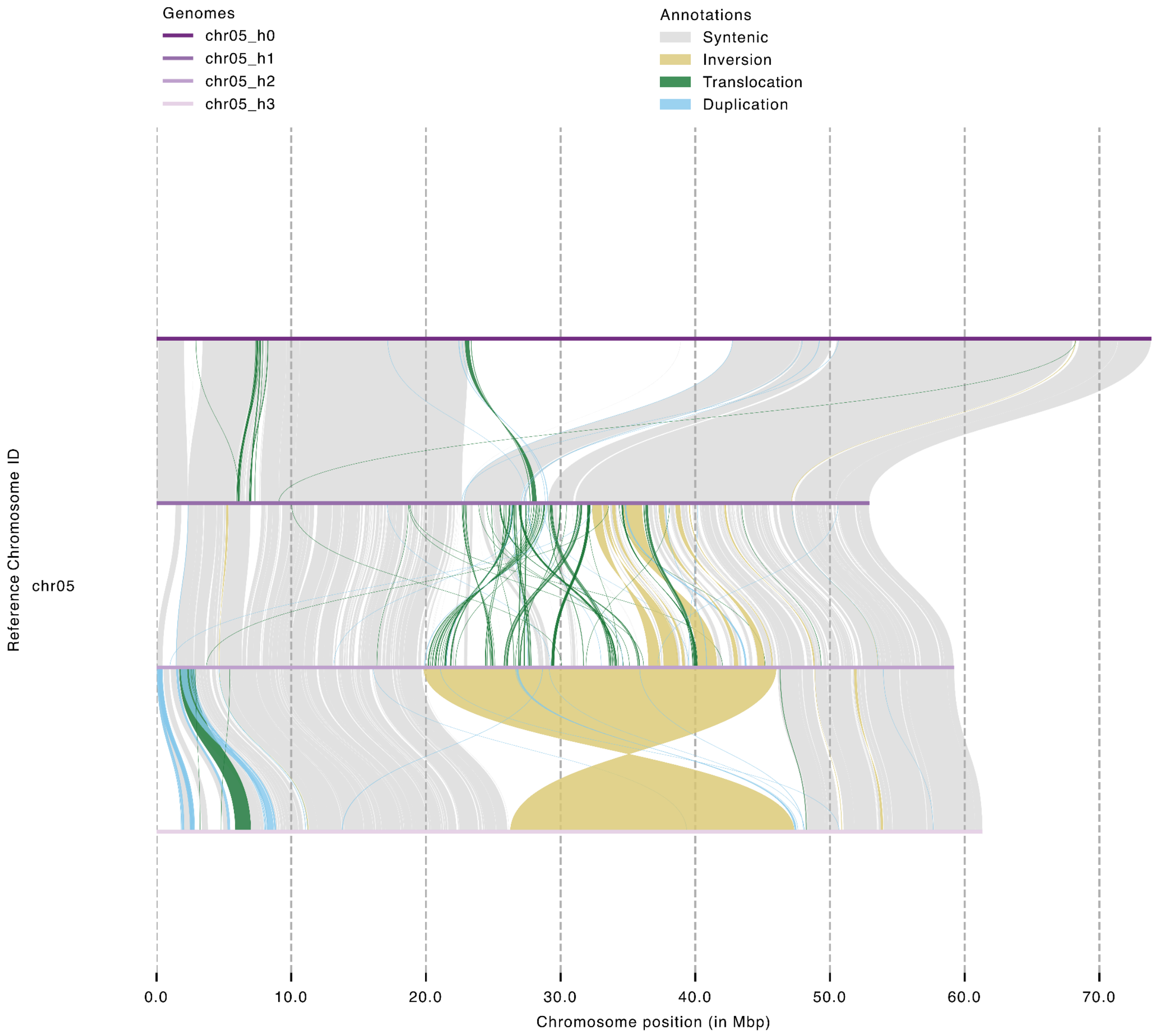

Fig. S21: Synteny analysis of chr05.

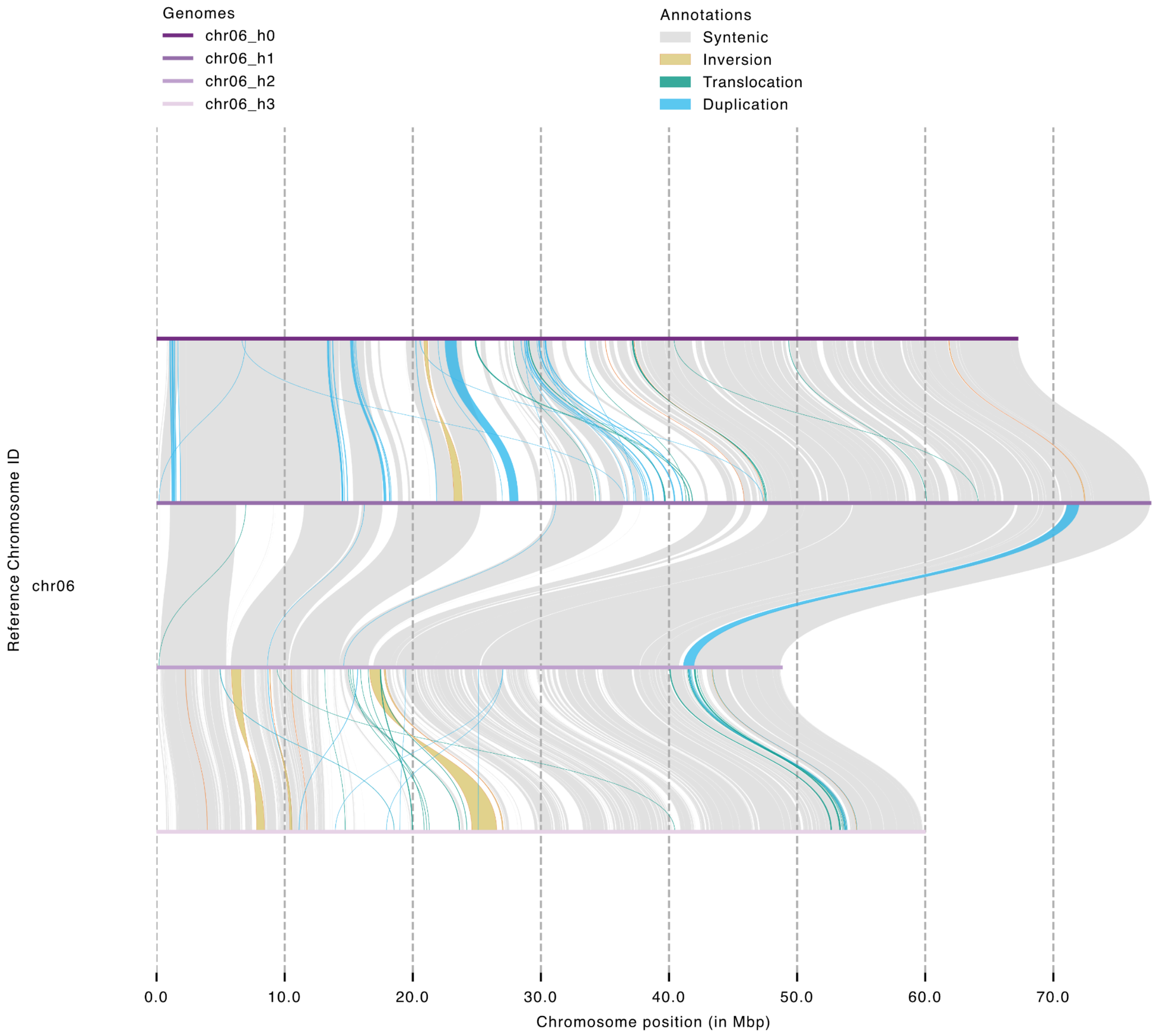

Fig. S22: Synteny analysis of chr06.

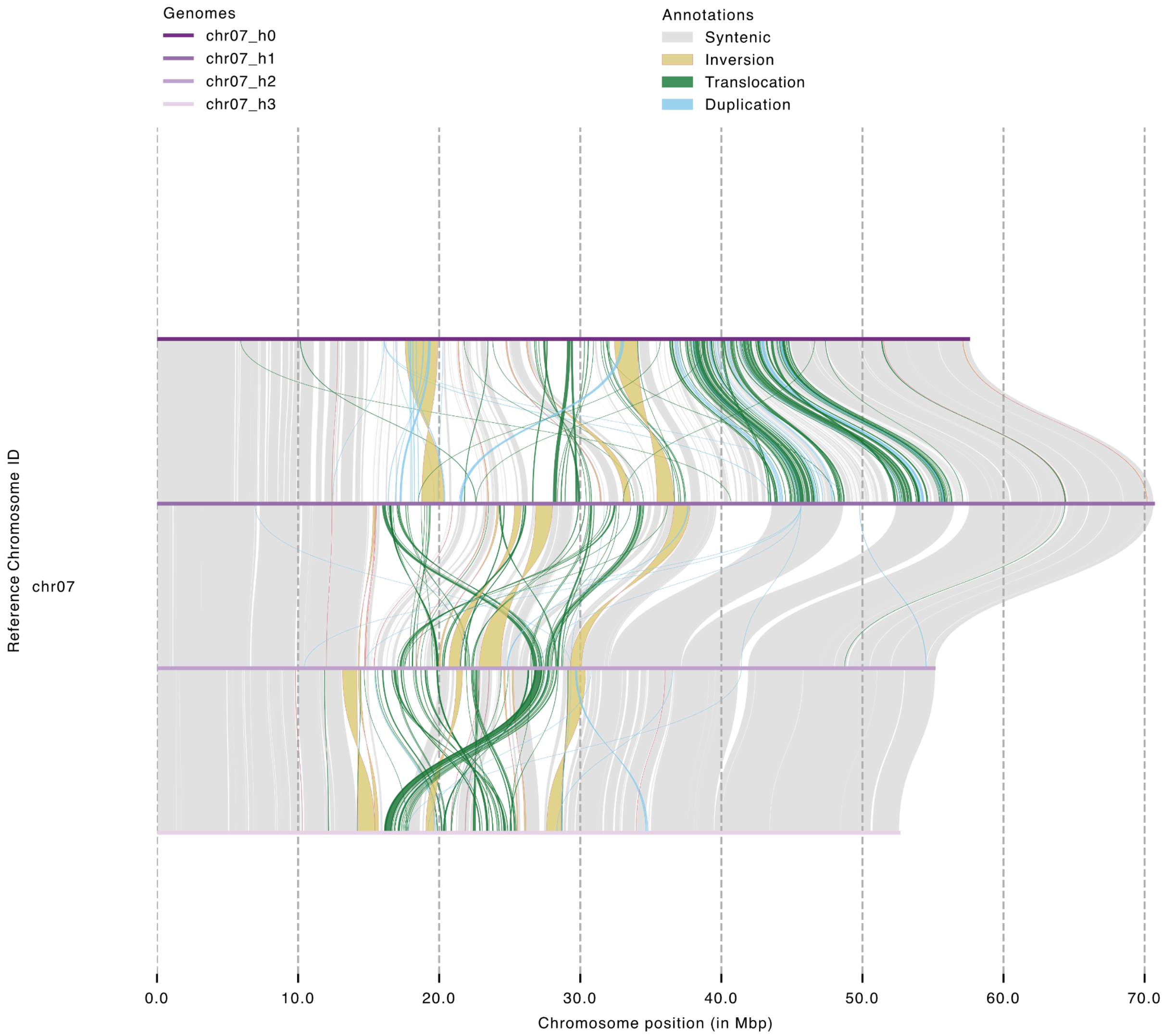

Fig. S23: Synteny analysis of chr07.

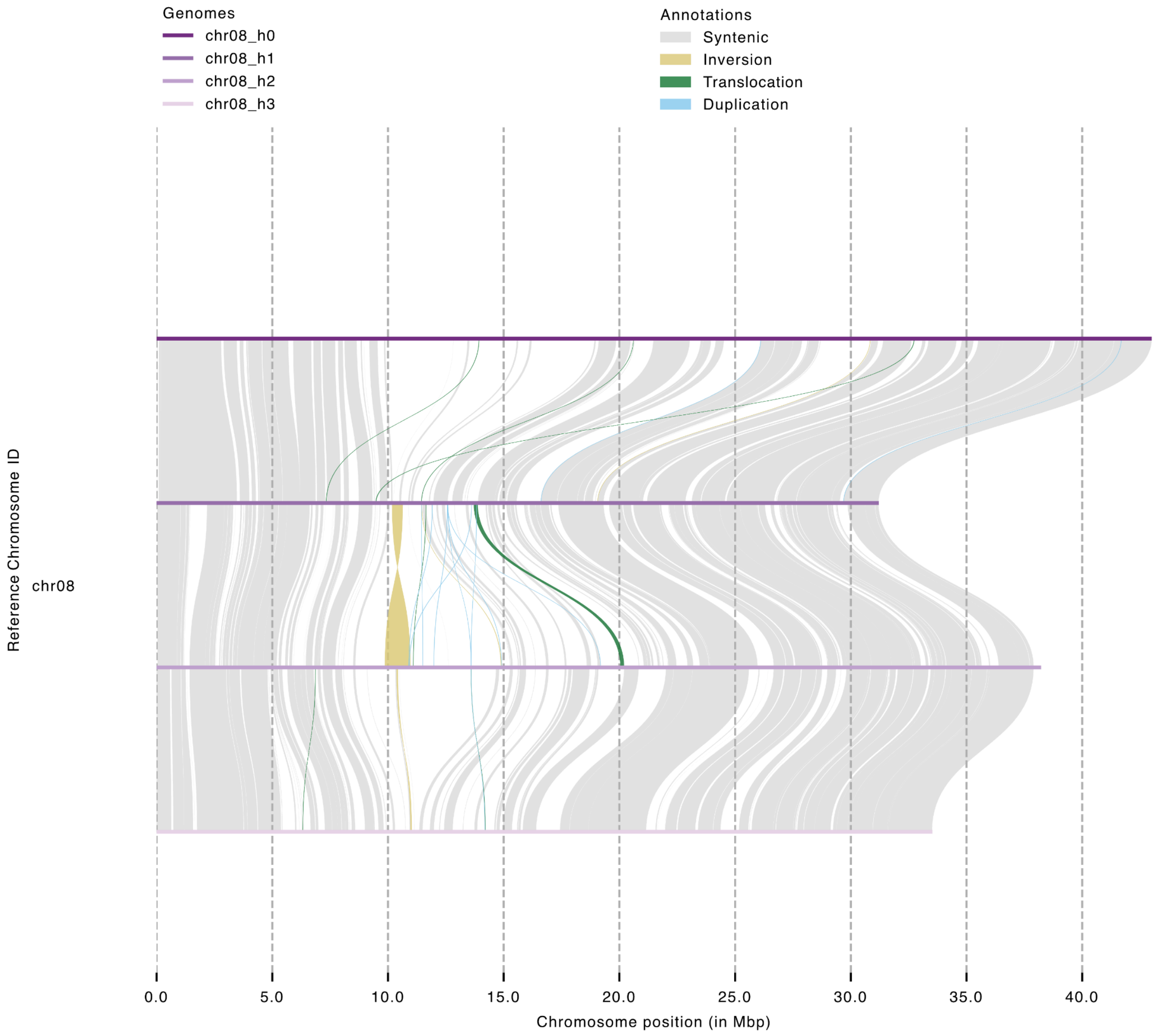

Fig. S24: Synteny analysis of chr08.

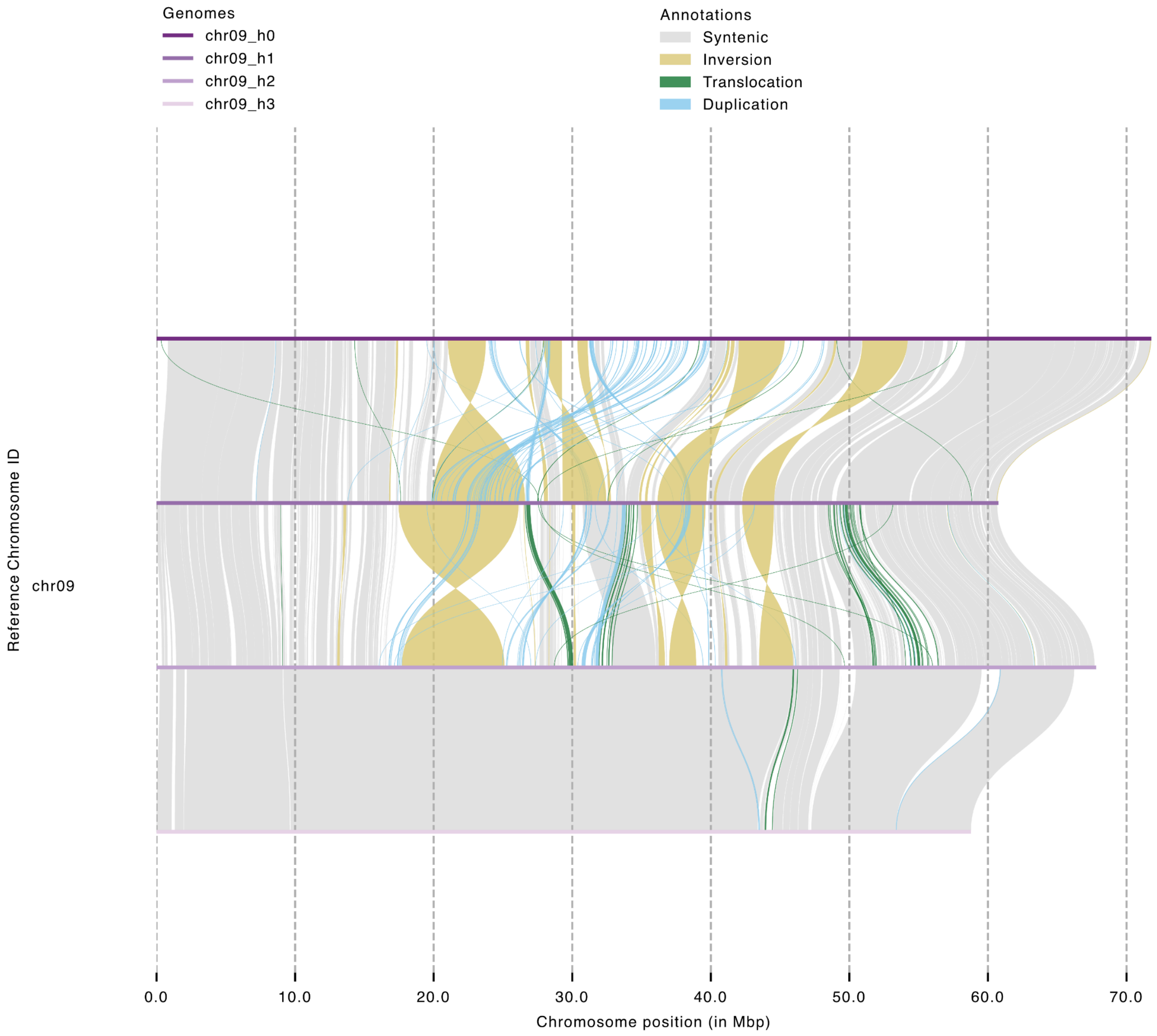

Fig. S25: Synteny analysis of chr09.

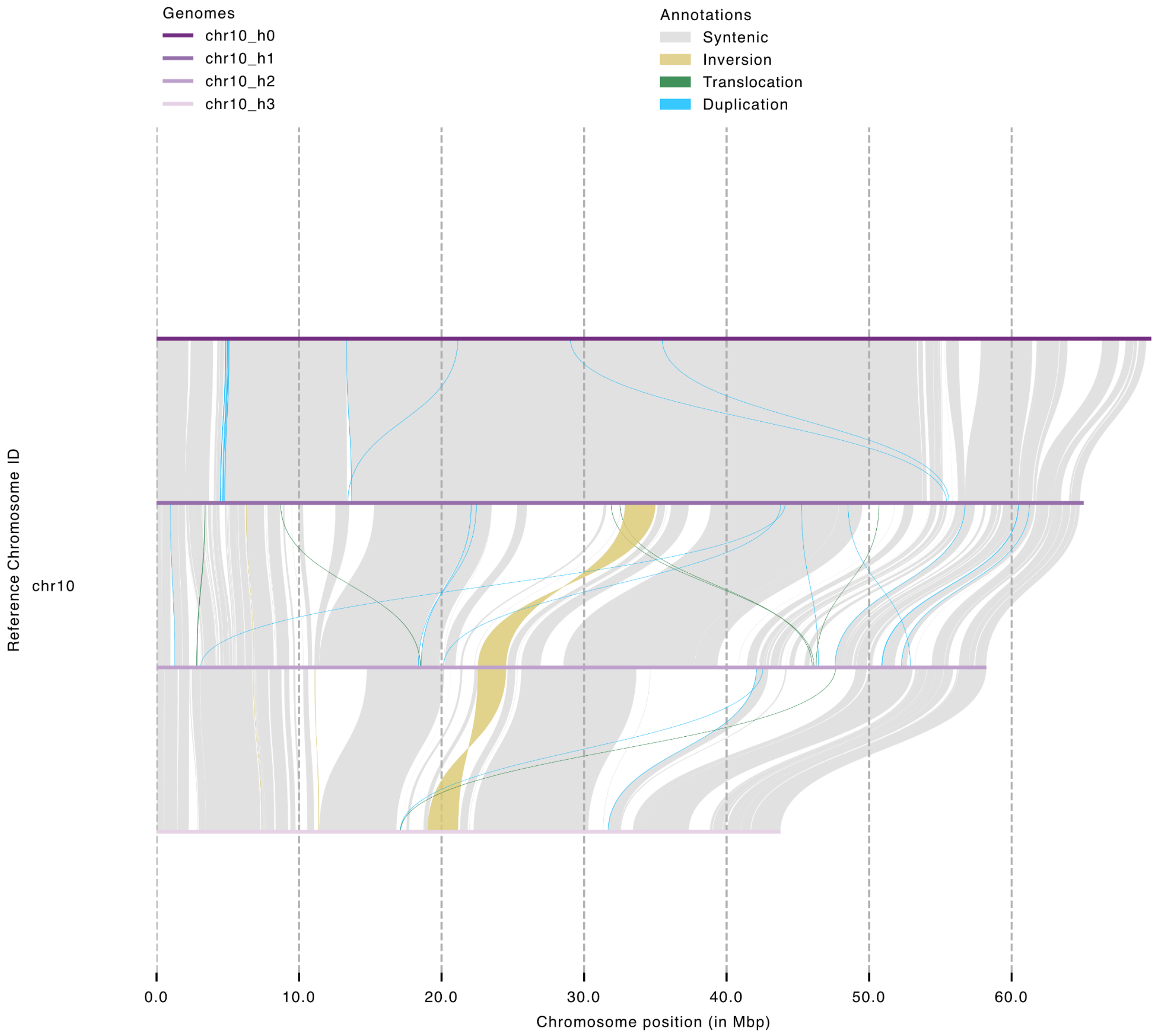

Fig. S26: Synteny analysis of chr10.

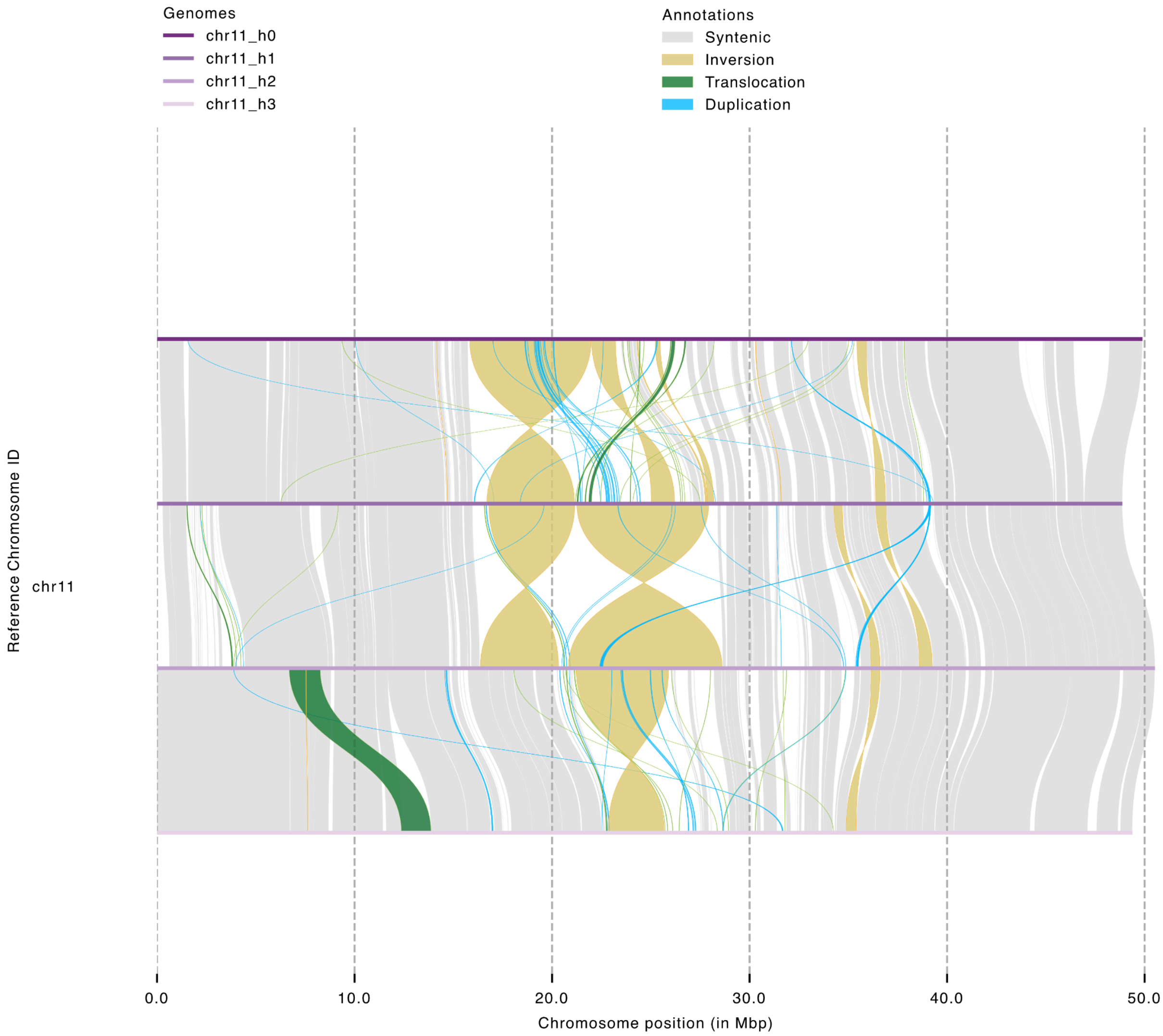

Fig. S27: Synteny analysis of chr11.

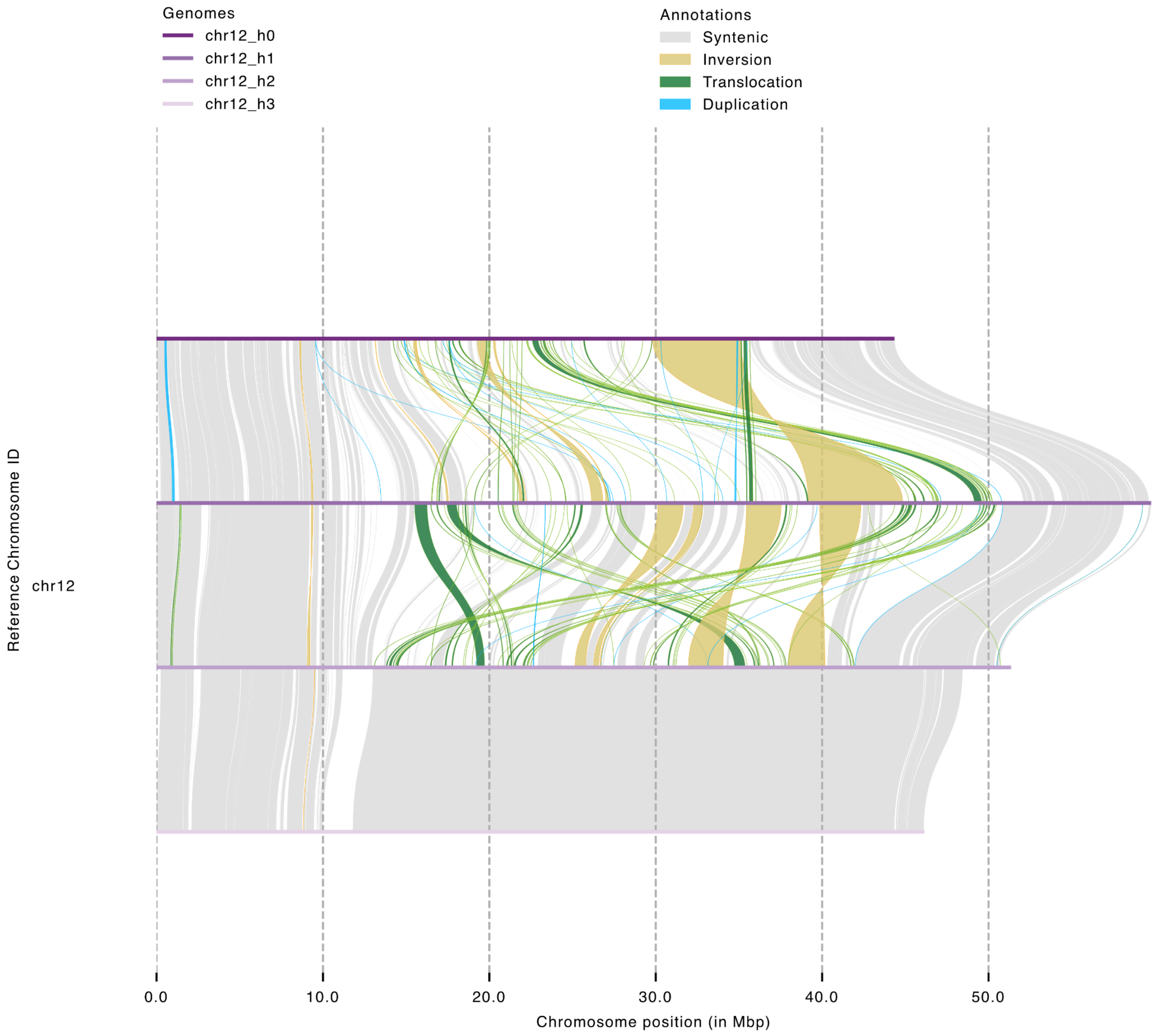

Fig. S28: Synteny analysis of chr12.

Pore-C data analysis for phasing validation

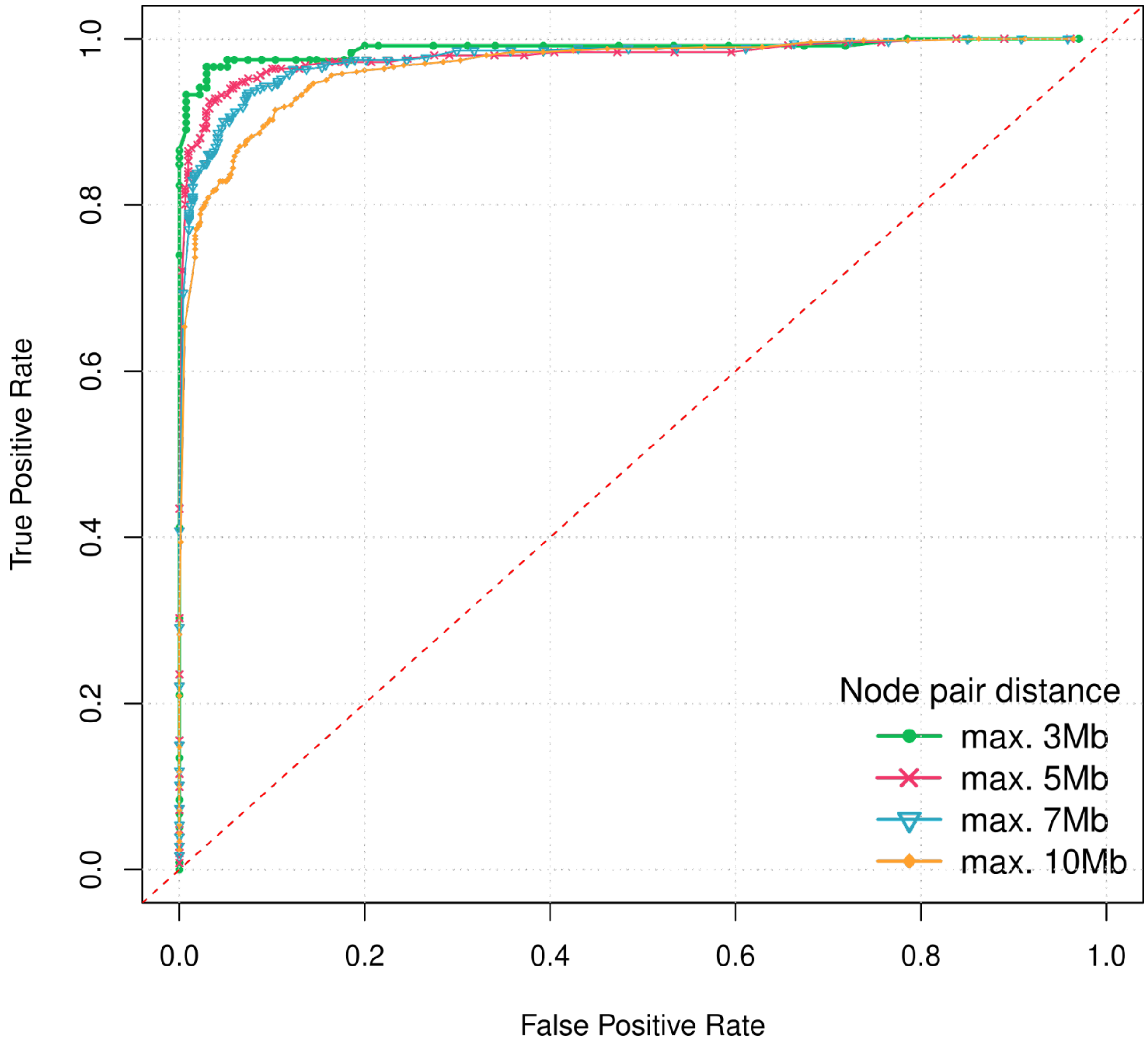

Fig S29: ROC curves for four different distance cutoffs. To compute the data points, all Pore-C coverage cutoffs between 0 and 70 (in steps of 1) were tested, as well as some higher cutoffs (ranging between 100 and 1200).
