# Supplementary material for: Haplotype-resolved assembly of a tetraploid potato genome using long reads and low-depth offspring data
Source: Genome Biol. 2024 Jan 19;25:26. doi: 10.1186/s13059-023-03160-z (PMC10797741; doi:10.1186/s13059-023-03160-z)
Supplement: Supplementary file 2 — Additional file 2: Review history. [file 13059_2023_3160_MOESM2_ESM.docx]

Review History

**First round of review**

**Reviewer 1**

**Were you able to assess all statistics in the manuscript, including the appropriateness of statistical tests used?**

Yes.

**Were you able to directly test the methods?**

No.

**Comments to author:**

Mari et al. presented a method for haplotype-resolved genome assembly of a tetraploid potato of cultivar 'Altus' using long reads, where the haplotype phasing is assisted by low-depth whole-genome sequencing of a few hundred of offspring genomes of 'Altus' × 'Colomba', i.e., a strategy similar to a recent publication in Molecular Plant by Bao et al..

While the idea is generally good, the performance evaluation on the method is not sufficient to show its accuracy and superiority, especially in haplotype phasing. Major and minor concerns are given as below.

Major

There are very few evidences in the main text supporting the conclusion that "Our approach generates high-quality assemblies...".

According to Fig. 4b, there are unexpected large variations in size of haplotype-specific chromosomes. For example, the first haplotype of ch02 is ~25 Mb larger than the other three, and the fourth haplotypes of ch07, ch10 and ch12 are up to ~70 Mb smaller than the other three, respectively. Other chromosomes have similar issues. Highly likely, these unexpected large differences were from errors in phasing haplotypes, e.g., joining of different haplotypes or splitting of the same haplotype.

There is a need to evaluate the accuracy in haplotype phasing in the final assembly using quantitative metrics. In addition, there is a need to improve the completeness of the final assembly. Currently, 12% or probably even more of the initially assembled contigs had to be discarded before the haplotype phasing process, which is worrisome.

For different parameter settings, there is also a lack of proper performance evaluation of the proposed method.

Specifically, at line 22-40 on page 13, the authors created several subsets of the 193 offsprings to investigate the size of offspring population on the performance of the method in haplotype phasing. First, why 12 is the expected number of clusters? As indicated at line 8-11 on the same page, there should be 48 clusters. Second, using the number of clusters to indicate the accuracy in haplotype phasing or the performance of the method is not informative. Even if the number of clusters is correct, it does not mean that there are no mis-joined contigs from different haplotypes within a single cluster, as mentioned earlier. It would be more intuitive to define a metric in haplotype phasing, which should measure how many haplotype-specific variants are correctly phased in each of the 48 clusters and how it is like overall. Then, draw the curve of the metric with the increased offspring population size. From this curve, one can estimate what is the optimal size of offspring population for the method to deliver the best accuracy in haplotype phasing. Besides the size of offspring population, it is not clear how sequencing depth of offsprings would influence the performance of the method. Other key parameters such as the size of k are interesting to investigate.

Minor

1. The abstract is over generalised, with which it is hard to see how effective the method is.

In page 1, there is redundancy in "...achieve chromosomal clustering and haplotype phasing..." and "individual chromosomes with haplotype-specific sequence resolution of whole chromosome arms", and this makes it confusing regarding whether the method can achieve chromosome-level haplotype phasing or chromosome-arm-level haplotype phasing.

2. In lines 20 and 26 on page 6, it is not clear what genome size was used to calculate 24x and 1.5x. The genome size of 'Altus' was not given.

3. The size of the initial assembly was 2798 Mb (line 38-39 on page 6). However, the authors found that there are overlaps in hifiasm graphs (line 12-18 on page 7). Does it mean that some genomic sequences can be carried by more than one unitig in the initial assembly? If so, how much of the 2798 Mb represented for unique genomic sequences, after excluding such overlaps? This would be important in evaluating the completeness of the final assembly.

4. Line 33-34 on page 7, the authors found that there were 6212 contigs solely overlapping both neighbouring nodes. Have the authors checked why such nodes exist if they do not provide more sequence information? And, how large are these omitted 6212 contigs in size?

5.Line 37-38 on page 7, for the 8290 nodes, how large are they in size and are these the only nodes used in the following phasing process?

6. Line 48-49 on page 7, why a k-mer size of 71 was selected? How does k-mer size affect the performance of the proposed method?

7. At line 37-38 on page 7, the authors found 8290 nodes that could be assigned with a dosage estimation. Then at line 39-42 on page 8, the authors found 10,784 nodes that were phase informative. Why 2494 nodes are phase informative but they could not be assigned with a dosage value? And, without a dosage information, how can they be used in phasing with the offsprings (line 56 page 8 to line 2 page 9)?

Related to this, please clarify out of the 20,216 nodes in the initial assembly, how many (in number) and how much (in size) were used in haplotype phasing? How much do they represent the original genome?

8. Fig. 2b, why those sequences < 100 kb were excluded from the visualisation of dosage estimation? A similar (supplementary) figure but with the full set of contigs should be provided as a comparison.

9. Line 2-7 on page 11, the sentence "we assumed.... of offspring samples." was not clear.

10. Line 19-22 on page 11. It is incorrect to say that the bimodal distribution can classify the contigs pairs on the same and on different haplotypes. Indeed, by checking the distribution of dots in Fig. 3a, there are three sub-distributions, the first around [0.5, 1.0], the second around (-0.1, +0.1), and the third around [-1, -0.1], where the second and the third were overlapping. It is the first and the third that can classify the contigs pairs on the same and on different haplotypes.

11. Fig. 3a, by "all node pairs", the authors meant how many nodes (with dosage 1), how large are they in size, and how many node pairs?

12. Line 43-46 on page 13. To get an accurate basis for evaluating the sequence completeness of the final assembly, genome size should be estimated for 'Altus'.

13. Line 3-5 on page 14. In Fig. 4a, what does "The contigs of each chromosome cluster are mapped..." mean? Does "each chromosome cluster" refer to four haplotype-specific chromosomes or one haplotype-specific chromosome?

There are 48 haplotype-specific chromosomes like given in Fig. 4b, forming four sets of haplotype-specific genomes. For each chromosome of DMv6.1, there should be four alignments from 'Altus', but Fig. 4a cannot reflect such information.

In addition, alignment of contigs of 'Altus' in Fig. 4a can only indicate contigs belonging to that chromosome (or linkage group), while it cannot reflect haplotype phasing accuracy among four sets of haplotype variants of the same chromosome.

Together with the large variations in chromosome sizes as shown by Fig. 4b, as mentioned earlier, the statement at line 6-8 on page 14 that "…supporting the robustness of our clustering process." could not be supported.

14. For Table 1, the sum on size of haplotigs does not directly support haplotype phasing. It only supports the linkage grouping of contigs to some degree.

In addition, how much of the initial assembly of 2798 Mb were kept in the final assembly of 2801.26 Mb? 88.15% as given in line 48-49 on page 8, or the size of 8290 nodes as given in 37-38 on page 7?

**Reviewer 2**

**Were you able to assess all statistics in the manuscript, including the appropriateness of statistical tests used?**

No.

**Were you able to directly test the methods?**

Yes.

**Comments to author:**

The authors presented a novel strategy for polyploidy haplotype assembly of autotetraploid potato cultivar Altus using PacBio Hifi reads and low-depth sequencing reads from offspring samples. Autopolyploidy genomes have multiple sets of same homologous chromosomes which make haplotype-resolved assembly much challenging. The main idea underline the proposed method is to separate haplotype contigs based on certain k-mer signatures in offspring sequences of cross two cultivars. By using 96-fold coverage of Hifi sequencing reads and about 1.5X per haplotype 193 offspring re-sequencing reads, the authors generated a haplotype-resolved assembly of the genome of potato cultivar Altus.

Firstly, it is a little unclear for me about the main selling point of this study. If the main point was about the assembly strategy, the readers might be expecting additional test or examples to make sure that this method could be applied to most of the other autopolyploidy genomes. If the main contribution was about the haplotype-resolved potato genome, they might need to add further comparisons and applications of this new genome.

I think it is a good idea about using offspring K-mer signature to phase haplotypes. Given the importance of the offspring data, I think the genetic distance of the crossed two cultivars could largely affect the K-mer count. However, in this case, what's the relationship between the two cultivars? Most importantly, were all of the sequenced offspring data mapped to the unitigs? Whether the reads from the alleles of Colomba forced mapping to the Altus alleles and potentially affecting the K-mer count? The authors also need to define the "unique K-mers" clearly.

In terms of the final assembly, it seems one of the haplotype in chr7, chr 10 and chr 12 exhibiting obviously shorter length than other three corresponding haplotypes (Fig 4). Is there any specific characteristics of these chromosomes?

In order to further indicating the robustness of the strategy, the authors might also need to provide some experimental validations of the correctness of the assembly.

**Authors Response**

**Point-by-point responses to the reviewers’ comments:**

Reviewer #1:

Mari et al. presented a method for haplotype-resolved genome assembly of a tetraploid potato of cultivar 'Altus' using long reads, where the haplotype phasing is assisted by low-depth whole-genome sequencing of a few hundred of offspring genomes of 'Altus' × 'Colomba', i.e., a strategy similar to a recent publication in Molecular Plant by Bao et al..

*Response: We thank the reviewer for bringing the recent work by Bao et al. to our attention. This was developed in parallel and we had not been aware of this. We now cite this work. While this work shares the idea of using offspring sequencing data to aid the phasing of a long read genome assembly, there are also important differences. Importantly, in Bao et al., 1034 offspring from a selfing population are used, while we use 193 samples from Colomba x Altus offspring. Exploring both strategies has complementary value for different study designs in our view.*

While the idea is generally good, the performance evaluation on the method is not sufficient to show its accuracy and superiority, especially in haplotype phasing. Major and minor concerns are given as below.

*Response: We thank the reviewer for the comments and now base additional evaluations on both Illumina and newly generated ONT data, as we describe in detail below.*

Major

There are very few evidences in the main text supporting the conclusion that "Our approach generates high-quality assemblies...". According to Fig. 4b, there are unexpected large variations in size of haplotype-specific chromosomes. For example, the first haplotype of ch02 is ~25 Mb larger than the other three, and the fourth haplotypes of ch07, ch10 and ch12 are up to ~70 Mb smaller than the other three, respectively. Other chromosomes have similar issues. Highly likely, these unexpected large differences were from errors in phasing haplotypes, e.g., joining of different haplotypes or splitting of the same haplotype. There is a need to evaluate the accuracy in haplotype phasing in the final assembly using quantitative metrics. In addition, there is a need to improve the completeness of the final assembly. Currently, 12% or probably even more of the initially assembled contigs had to be discarded before the haplotype phasing process, which is worrisome.

*Response: Thank you for bringing up these observations. Indeed, the reason for some of the haplotypes exhibiting shorter length as their counterparts did not become clear from the figure. In fact, this was due to parts that remained unphased during the haplotype clustering, not due to errors in phasing haplotypes. We were now able to investigate on this further and to resolve this issue. Because of the rather conservative nature of our clustering procedure to avoid misassemblies, several contigs were not added to a haplotype cluster and remained as unphased, which led to one haplotype being considerably shorter, as it was missing these contigs. We now employ a postprocessing step in which we review the remaining unphased nodes and assigned them to the cluster with the highest correlation if a clear result emerges. We have updated the figure accordingly, showing that the haplotypes of ch07, ch10 and ch12 - which were formerly problematic - now have assimilated in length.*

*We also want to emphasise that contigs that could not be clustered do not need to be completely discarded. They contain valid sequence but not enough unique k-mers to be reliably assigned. We realised that this was not sufficiently clear in our previous figure. Therefore, we have added the information to the plot how much sequence was included in the first clustering step to form the chromosomal cluster, but had to be excluded from the second step of phased clustering due to not being phase-informative (= lacking the unique k-mer information). As can be seen in Figure 4b (pasted below), this "unphased" portion (light grey) is higher in chromosomes 4, 8 and 10, while e.g. chromosomes 7 and 11 are rather complete.*

*Another factor contributing to the former haplotype size differences in ch10 and ch12 was the following: As highlighted in our later discussion in the paper, we identified a mix-up during the initial chromosomal clustering, leading to the assignment of a problematic component containing parts of two chromosomes to both haplotypes. This mix-up was attributed to an error in hifiasm, incorrectly joining parts of chromosomes 10 and 12 on the same graph component. Consequently, we now excluded these nodes from the clustering analysis to avoid these misassignments.*

*Concerning ch02, one haplotype still appears larger than expected. However, we have carefully examined this issue and presented a corresponding plot in the supplementary material to demonstrate that it is unlikely to be a misjoin with sequence from another chromosome. The nodes from this haplotype indeed map specifically to ch02 in the reference. We have addressed this finding in the manuscript.*

*Updated Figure 4b: Length comparison of the four haplotypes (grey bars) compared to the reference (coloured bars). The length is computed as the sum of the contig lengths for all contigs in a haplotype cluster. We have added the information how much node sequence was included in the first clustering step to form the chromosomal cluster, but had to be excluded from the second step of phased clustering due to not being phase-informative (= lacking the unique k-mer information), marked by the light grey bars atop of each cluster size bar. This visualisation distributes the unphased sequence uniformly to each haplotype cluster. Note that the true assignment to haplotypes is, by definition, unknown for the unphased sequence.*

*New Suppl. Fig 6: Ch02 haplotype alignments to the DMv6.1 reference, showing no relevant mappings to other chromosomes.*

*To support our findings, we have applied several methods for assembly evaluation. We make use of Illumina sequencing data of Altus (2x150bp paired-end reads) to apply a combination of meryl and Merqury, which evaluate assembly qualities based on k-mer spectra in the Illumina read sets as well as the assembly. This constitutes a reference-free evaluation method for assessing assembly quality.*

*Our Merqury results are as follows: For the assessment of assembly completeness, 751,364,022 k-mers (k=21) were found in the resulting assembly, while in the read set, a total of 839,743,760 k-mers was present, leading to a completeness value of 89.4754%. This value refers to those nodes which are both clustered and phased to a haplotype. When taking into account all nodes that are present in a cluster after the chromosomal clustering step, the completeness value increases to 97.93%.*

*For the QV estimation, we obtained the following results: 1,500,790 unique k-mers were found in the Altus assembly, 2,636,267,980 k-mers were in total present in both the assembly and the read set. The resulting QV value was 45.6677, equivalent to an error rate of 2.71162e-05 or one error every 36,878 base pairs on average.*

*To estimate the completeness of our assembly in terms of expected gene content, we applied BUSCO using the solanales_odb10 database and received a completeness score of 96.8%, which is in line with the completeness scores listed for the DMv6.1 reference (97.9), the assembly of C88 (Bao et al., 96.28%) and the assembly of Otava (Sun et al., 97.3%).*

*Additionally, we have generated new sequencing data for Altus using the ONT long read platform. We used these data to validate the assignment of dosage estimates, which is difficult to do from short reads. To this end, we repeated the initial coverage analysis using the ONT data and compared dosage estimates found for each node based on HiFi to those based on ONT. The joint distribution of ONT- and HiFi-based coverage estimates can be found in the new Supplementary Figure 3 and is discussed in the manuscript in Section “Dosage Analysis”.*

*Suppl. Fig 3: Joint coverage of ONT reads as well as HiFi reads, mapped to the hifiasm assembly graph of Altus. a: All nodes without any filtering applied, b: Filtered to only show coverage of nodes with a unique sequence length of at least 100kb.*

For different parameter settings, there is also a lack of proper performance evaluation of the proposed method.

Specifically, at line 22-40 on page 13, the authors created several subsets of the 193 offsprings to investigate the size of offspring population on the performance of the method in haplotype phasing. First, why 12 is the expected number of clusters? As indicated at line 8-11 on the same page, there should be 48 clusters.

*Response: 12 is the expected number of clusters since in the first step, our clustering method determines the chromosome clusters, of which we expect 12. It is only in the second step that these clusters are further refined to specific haplotype clusters. In our experiments with offspring sample numbers lower than 150, we noticed that even the 12 chromosomal clusters were not distinguished properly. Without this step, it is not useful to carry on any further phasing steps. We now clarified this in the text.*

Second, using the number of clusters to indicate the accuracy in haplotype phasing or the performance of the method is not informative. Even if the number of clusters is correct, it does not mean that there are no mis-joined contigs from different haplotypes within a single cluster, as mentioned earlier. It would be more intuitive to define a metric in haplotype phasing, which should measure how many haplotype-specific variants are correctly phased in each of the 48 clusters and how it is like overall. Then, draw the curve of the metric with the increased offspring population size. From this curve, one can estimate what is the optimal size of offspring population for the method to deliver the best accuracy in haplotype phasing.

*Response: Thank you for your suggestion regarding the use of a more informative metric to evaluate the accuracy of our haplotype phasing method. We agree that this would be a great way to show phasing accuracies. However, at this stage, we do not have any reliable method to obtain haplotype-specific variants for the sample analysed in our study other than our own haplotype assembly method; e.g, there is no parent data of Altus available. In the absence of haplotype-specific variants, we have found that the number of clusters is a useful indicator of the accuracy of our results, as it reflects the ability of our method to correctly identify and separate different haplotypes. We have also created an extended version of Fig. 4a as a new supplementary figure which showcases for each chromosome the four single haplotypes, resulting in a total of 48 mappings to the reference, for a more informative visualisation.*

*New Suppl. Fig 7: Haplotype-resolved version of main Fig. 4a to show the mappings of all single haplotypes to the reference.*

Besides the size of offspring population, it is not clear how sequencing depth of offsprings would influence the performance of the method.

*Response: Unfortunately, we do not have additional sequencing data from the offspring samples with increased sequencing depth. While we agree that it would be interesting to explore how a higher offspring coverage influences the performance of our method, we cannot address this question in the current study. On the other hand, the coverage is already low with 1.5x per haplotype. We think that this is already on the lower boundary of useful coverage values, so that further downsampling the coverage to even lower values does not appear to be a common use case.*

Other key parameters such as the size of k are interesting to investigate.

*Response: During our initial parameter exploration, we considered smaller sizes of k, such as k=31 and k=51. However, smaller k values would have exacerbated the issue of unitigs lacking unique k-mers, further compromising the completeness of the phasing process. For k=51, we would lose 394 unitigs and for k=31, we would lose 1047 unitigs. Taking this into consideration, we made the decision to prioritise the parameter value of k=71, as it offered the highest number of unitigs with unique k-mers compared to the previously tested values. On the other hand, given the short length of the offspring Illumina reads (2x150bp paired-end reads), larger values of k do not make much sense.*

Minor

1. The abstract is over generalised, with which it is hard to see how effective the method is.

In page 1, there is redundancy in "...achieve chromosomal clustering and haplotype phasing..." and "individual chromosomes with haplotype-specific sequence resolution of whole chromosome arms", and this makes it confusing regarding whether the method can achieve chromosome-level haplotype phasing or chromosome-arm-level haplotype phasing.

*Response: First, we’d like to note that the abstract has a very strict limit of 100 words, and we had to shorten a previous, more nuanced, version considerably. With the term “Chromosomal clustering and haplotype phasing”, we refer to: “clusters of chromosomes are formed, and haplotypes are phased within.” With the term “[..] chromosomes with haplotype-specific sequence resolution of whole chromosome arms”, however, we refer to the result of the chromosomal clustering + haplotype phasing. We have now slightly rephrased the last sentence of the abstract to remove ambiguity and hope it is more clear now.*

2. In lines 20 and 26 on page 6, it is not clear what genome size was used to calculate 24x and 1.5x. The genome size of 'Altus' was not given.

*Response: See our response to point 12, where we estimate the genome size.*

3. The size of the initial assembly was 2798 Mb (line 38-39 on page 6). However, the authors found that there are overlaps in hifiasm graphs (line 12-18 on page 7). Does it mean that some genomic sequences can be carried by more than one unitig in the initial assembly? If so, how much of the 2798 Mb represented for unique genomic sequences, after excluding such overlaps? This would be important in evaluating the completeness of the final assembly.

*Response: Hifiasm graphs always contain overlaps between the nodes. Therefore, those sequence parts that lie on such an overlap are by definition carried by the two unitigs that contain the overlapping sequence. We restrict our analysis to include only the unique sequences when estimating the dosages of a node, and for phasing as well (by design, since we use unique k-mers). In total, 2.217 Gb are represented by the sum of non-overlapping sequences of all nodes in the initial assembly. In this revision, we have added a Merqury evaluation using Illumina sequencing data. This results in completeness estimates that are based on a comparison of the k-mer spectra in the assembly vs the Illumina reads, which is not affected by any overlaps between unitigs.*

4. Line 33-34 on page 7, the authors found that there were 6212 contigs solely overlapping both neighbouring nodes. Have the authors checked why such nodes exist if they do not provide more sequence information? And, how large are these omitted 6212 contigs in size?

*Response: We are not aware of any particular reason why hifiasm creates such nodes in some cases. This is however not restricted to this specific study, but occurs more frequently. The lengths of the nodes are also quite short: the longest node was 42,105 bp (0.148 Gb in total across all such nodes). We have added this information to the main text.*

5.Line 37-38 on page 7, for the 8290 nodes, how large are they in size and are these the only nodes used in the following phasing process?

*Response: Similar to what we explain in point 7: At the time of submission, regarding the first version of our assembly method, the nodes used in the phasing process were all the phase informative nodes. Now, the nodes used for phasing 1) are phase-informative, i.e. they contain unique k-mer information and 2) have the same dosage estimates based on both the HiFi and the ONT data. This use of ONT data is optional, but we opted to include it here to start from the nodes with the most reliable dosage estimates. The 8290 nodes we talked about made up 2.396 Gb. The 6982 nodes where the dosage estimates of HiFi and ONT reads are equal make up 2.233 Gb.*

6. Line 48-49 on page 7, why a k-mer size of 71 was selected? How does k-mer size affect the performance of the proposed method?

*Response: See our response to Point 4 (‘major’) above.*

7. At line 37-38 on page 7, the authors found 8290 nodes that could be assigned with a dosage estimation. Then at line 39-42 on page 8, the authors found 10,784 nodes that were phase informative. Why 2494 nodes are phase informative but they could not be assigned with a dosage value? And, without a dosage information, how can they be used in phasing with the offsprings (line 56 page 8 to line 2 page 9)?

*Response: 10,784 nodes are phase informative, which means that they contain unique k-mers, which is essential for the clustering process. This is not directly related to the dosage information. 2494 nodes have an estimated dosage of ‘0’ due to low depth, which in turn is due to the filtering of all alignments with a mapping quality below 60. For the phasing process, these nodes were formerly included nonetheless (with dosage 1) in case they contain unique k-mers, i.e. are phase informative.*

*For the revised assembly, we have now chosen a more restrictive process of determining the dosage information since we additionally sequenced ONT data of Altus and make use of these reads to also estimate the dosages independent of the HiFi estimation. We now include only those phase-informative nodes (= nodes having unique k-mers) which at the same time showed an equal dosage estimate in the ONT and the HiFi data, for a safer estimation of dosages, which is a crucial step for the phasing process and therefore adds to the accuracy of the results - of course at the expense of slightly fewer sequence that can be used during the clustering: The resulting set consists of 6982 nodes with a total length of 2.233Gb, which is 93.22% of the 2.396Gb that consisted of the 8290 nodes we initially assigned dosages to.*

*For a visualisation of the different node sets introduced throughout our study, see the UpSet plot in the new Supplementary Fig. 4 (below):*

*Suppl. Fig 4: UpSet plot of the various node sets used throughout this study. ‘Node’ refers to the unitigs from the initial assembly graph. ‘Over_100kb’: nodes with a length of at least 100 kb. ‘Dosage’: Nodes for which we obtained dosage information, i.e. the dosage estimate of HiFi and ONT reads coincides. ‘Unique_kmers’: Nodes that contain >0 unique k-mers. ‘Unique_sequences’: Nodes for which the unique – i.e. the non-overlapping – sequence is longer than 0bp.*

Related to this, please clarify out of the 20,216 nodes in the initial assembly, how many (in number) and how much (in size) were used in haplotype phasing? How much do they represent the original genome?

*Response: Previously, 10,784 nodes (= all the phase informative nodes) were used for the clustering process, in size 2.466 Gb, representing 88.15% of the genome. This is according to what we previously described on page 8. Now, we are using the restricted set of 6982 nodes with a total length of 2.233Gb. However, we want to emphasise that the final step of creating haplotype traversals can (and will) involve nodes not part of the initial phasing step, i.e. nodes where the phase assignment can be inferred from the graph topology and the assignment of neighbouring nodes will be assigned to a haplotype. That is reflected in a final phased assembly length of 2.78Gbp.*

8. Fig. 2b, why those sequences < 100 kb were excluded from the visualisation of dosage estimation? A similar (supplementary) figure but with the full set of contigs should be provided as a comparison.

*Response: As mentioned on page 7 in the paragraph “Dosage analysis”, the requested figure is already presented in the supplement: Supplementary Figure 2 shows the same dosage distribution plot, but without the restriction of a unique sequence length of at least 100kb. The only filtering presented in Suppl. Fig. 2 is for contigs with an exceptionally low coverage for clarity of visualisation. However, we have added a second plot to Suppl. Fig. 2 without the filtering for coverage.*

9. Line 2-7 on page 11, the sentence "we assumed.... of offspring samples." was not clear.

*Response: We rephrased the sentence to make it more clear. New sentence: “For our correlation-based approach, we exploit that contigs from the same haplotype have similar k-mer count patterns because the corresponding haplotype context is transmitted to the same subset of offspring samples.”*

10. Line 19-22 on page 11. It is incorrect to say that the bimodal distribution can classify the contigs pairs on the same and on different haplotypes. Indeed, by checking the distribution of dots in Fig. 3a, there are three sub-distributions, the first around [0.5, 1.0], the second around (-0.1, +0.1), and the third around [-1, *-0.1], where the second and the third were overlapping. It is the first and the third that can classify the contigs pairs on the same and on different haplotypes.*

*Response: We did not claim that one can predict the haplotype status (cis vs trans) for each pair of nodes from their correlation value alone. While the signal is fairly strong for short genomic distances, the power to cluster by haplotype comes from looking at many node pairs in turn as part of our agglomerative clustering procedure. But we acknowledge the confusion the last sentence of that paragraph could have caused and have removed the claim of a bimodal*

11. Fig. 3a, by "all node pairs", the authors meant how many nodes (with dosage 1), how large are they in size, and how many node pairs?

*Response: The 4,830 dosage 1 nodes of the largest components account for 947.78 Mb. After removing pairs which had no valid correlation (NaN), 701,582 pairs remained in the dataset for plotting. We have added this to the caption of Fig. 3a.*

12. Line 43-46 on page 13. To get an accurate basis for evaluating the sequence completeness of the final assembly, genome size should be estimated for 'Altus'.

*Response: We now address assembly completeness in two ways. First, using the Illumina sequencing data of Altus (SRA accessions SRR14993639 and SRR14993640), we employed Merqury for evaluation which results in a completeness estimate of 97.93%. Furthermore, we used GenomeScope2.0 to estimate the haploid genome size from the PacBio Hifi reads, yielding a value of 730.6 Mb (see new Supplementary Figure 5 below), which is both consistent with the size of DMv6.1 (731.3Mb) and results in a similar completeness estimate (2.78Gbp of phased assembly vs. 4*730.6Mbp) of 95.1%.*

*Suppl. Fig 5: Genome size estimation using GenomeScope.*

13. Line 3-5 on page 14. In Fig. 4a, what does "The contigs of each chromosome cluster are mapped..." mean? Does "each chromosome cluster" refer to four haplotype-specific chromosomes or one haplotype-specific chromosome?

*Response: It refers to all four haplotype-specific clusters per chromosome.*

There are 48 haplotype-specific chromosomes like given in Fig. 4b, forming four sets of haplotype-specific genomes. For each chromosome of DMv6.1, there should be four alignments from 'Altus', but Fig. 4a cannot reflect such information.

*Response: We agree, and as described above, we have added a supplementary figure showing the alignments of all 48 chromosomes (Supplementary Figure 7).*

In addition, alignment of contigs of 'Altus' in Fig. 4a can only indicate contigs belonging to that chromosome (or linkage group), while it cannot reflect haplotype phasing accuracy among four sets of haplotype variants of the same chromosome.

*Response: This is correct, and we made sure to indicate this in the manuscript. We make no claims that this figure is indicative of haplotype phasing accuracy.*

Together with the large variations in chromosome sizes as shown by Fig. 4b, as mentioned earlier, the statement at line 6-8 on page 14 that "…supporting the robustness of our clustering process." could not be supported.

*Response: We clarified in the text that this statement refers to the chromosome clustering process. Additionally, note that in the revised assembly with all additional measures taken, the large variations in chromosome sizes have been resolved (as described above) and the lengths of the sequences that could not be assigned to a haplotype are now additionally displayed in the figure.*

14. For Table 1, the sum on size of haplotigs does not directly support haplotype phasing. It only supports the linkage grouping of contigs to some degree.

In addition, how much of the initial assembly of 2798 Mb were kept in the final assembly of 2801.26 Mb? 88.15% as given in line 48-49 on page 8, or the size of 8290 nodes as given in 37-38 on page 7?

*Response: In the final phased assembly, 2.31Gb of nodes from the initial assembly was kept. After the chromosome clustering, i.e. unphased nodes can be contained in the set, 2.69Gb of nodes from the initial assembly are kept. Note that due to our threading step after the haplotype clustering, nodes that are phase-uninformative and as such unphased may be recovered and added to the corresponding haplotig. Therefore, the final assembly is not limited to phase-informative nodes only.*

Reviewer #2:

The authors presented a novel strategy for polyploidy haplotype assembly of autotetraploid potato cultivar Altus using PacBio Hifi reads and low-depth sequencing reads from offspring samples. Autopolyploidy genomes have multiple sets of same homologous chromosomes which make haplotype-resolved assembly much challenging. The main idea underline the proposed method is to separate haplotype contigs based on certain k-mer signatures in offspring sequences of cross two cultivars. By using 96-fold coverage of Hifi sequencing reads and about 1.5X per haplotype 193 offspring re-sequencing reads, the authors generated a haplotype-resolved assembly of the genome of potato cultivar Altus.

Firstly, it is a little unclear for me about the main selling point of this study. If the main point was about the assembly strategy, the readers might be expecting additional test or examples to make sure that this method could be applied to most of the other autopolyploidy genomes. If the main contribution was about the haplotype-resolved potato genome, they might need to add further comparisons and applications of this new genome.

*Response: This is a “Method” article as per the Genome Biology article types. Still, our manuscript presents both a method for polyploid genome assembly and the resulting assembly of the Altus genome. While we have put more emphasis on our method, we are also confident that our assembly poses a useful addition to the - at present still few - phased potato assemblies. Especially in the light of pangenomics, having another cultivar assembled - and in partial haplotype resolution - can be a useful contribution. Owing to the method focus of our article, we do not develop this direction further in this article. No part of our method is restricted to potato genomes or to tetraploid genomes in general. In fact, any setting of a (diploid or polyploid) genome where high quality reads and additionally sequencing data for a larger set of offspring is available, can be applied here. However, we have to restrict our analysis in this paper to the potato genome, given the data that we have currently available.*

*To address the reviewer’s comment, we have added different quantitative analyses to the paper and compared our assembly to other existing potato assemblies. For example, we have computed BUSCO scores and got a completeness score of 96.8%. In comparison, this is similar to the completeness score listed for the DMv6.1 reference (97.9%), the assembly of C88 (Bao et al., 96.28%) and the assembly of Otava (Sun et al., 97.3%). Regarding repeat content, we are also seeing similar results: We have annotated repeats using RepeatModeler and RepeatMasker and found 66.81% of repetitive content, the largest part of which was made up of LTRs. This is also in line with what was detected by Sun et al. in the assembly of Otava (66% of repetitive content, LTRs being the most abundant group) and by Pham et al. for the DMv6.1 assembly (66.8%). We did not find any estimation of repeat content in the publication of the C88 genome. Furthermore, we have applied reference-free assembly quality evaluation by Merqury, resulting in a QV value of 45.6677. This is similar to the QV value of C88 (46.6). Sun et al. report a slightly better QV value of 51.7 for Otava. For the phased result (i.e. taking into account only the fully phase-resolved parts of the haplotigs) Merqury estimates a completeness of 89.48%, while the result from the clustering (i.e. contigs that have been clustered into the chromosome, but might lack accurate phasing information due to, e.g., insufficient k-mer information) is 97.93%. In comparison, for Otava, a completeness score of 97.3% was reported, for C88 99.05%. We expect these differences, as our method utilises unique k-mers, which poses a challenge in genomes as repeat-rich as the potato genome. Also, since we use sequencing data from an offspring of the cross Altus x Colomba, we had to discard a number of k-mers which were also present in the Colomba genome to make sure that only the signal from Altus is taken into account.*

I think it is a good idea about using offspring K-mer signature to phase haplotypes. Given the importance of the offspring data, I think the genetic distance of the crossed two cultivars could largely affect the K-mer count. However, in this case, what's the relationship between the two cultivars?

*Response: We have checked the Potato Pedigree Database (van Berloo et al., 2007, http://www.plantbreeding.wur.nl/PotatoPedigree/) for the evolutionary background of the Altus cultivar. The maximum number of generations that can be detected in the ancestry of the cultivar is 8. In these last 8 generations, Colomba did not appear on the pedigree of Altus and vice versa. (The pedigree for Altus is too large to paste here, but can be seen at https://www.plantbreeding.wur.nl/PotatoPedigree/pedigree_imagemap.php?id=824&showjaar=0 &depth=8). Considering this and also taking into account that Colomba is a consumption potato, while Altus is a starch variety (so the purposes of the two cultivars are very different), it is safe to conclude that the two cultivars are not closely related to one another. How different divergence rates influence the results is an interesting question for future research, but presently we do not have access to more data sets to test this. While this needs to be demonstrated experimentally in future research, we expect that the process is robust to both varying rates of between-cultivar and within-cultivar (between haplotype) divergence. We anticipate that the most challenging scenario would be one where the heterozygosity is so low that neighbouring heterozygous sites are so far apart that they are not on the same long reads and that there is a significant chance of recombination between them. But that degree of homozygosity would be extremely unusual in plants (and would be a situation where phasing is almost unnecessary).*

Most importantly, were all of the sequenced offspring data mapped to the unitigs? Whether the reads from the alleles of Colomba forced mapping to the Altus alleles and potentially affecting the K-mer count? The authors also need to define the "unique K-mers" clearly.

*Response: There seems to be a misunderstanding here. We would like to clarify that no offspring data were mapped to the unitigs in our study. Instead, we used the assembly graph unitigs to identify unique k-mers in Altus, which were then counted in the offspring sequencing data. The result of this counting procedure is used for haplotype phasing of Altus. In particular, we defined 'unique k-mers' as 'k-mers that exist uniquely (= exactly once)’ in the assembly graph (page 8, line 9) and additionally, do not exist in the Colomba genome. This definition was used consistently throughout the manuscript to refer to k-mers that were specific to the Altus genome and for the respective unitig they occurred on. On page 8, we have now clarified the definition of unique k-mers and hope this becomes clearer now. Therefore, there is no possibility of the reads from the alleles of Colomba affecting the k-mer count and, in turn, the haplotype phasing results. We hope that this explanation makes things clearer.*

In terms of the final assembly, it seems one of the haplotype in chr7, chr 10 and chr 12 exhibiting obviously shorter length than other three corresponding haplotypes (Fig 4). Is there any specific characteristics of these chromosomes?

In order to further indicating the robustness of the strategy, the authors might also need to provide some experimental validations of the correctness of the assembly.

*Response: This coincides with what Reviewer 1 has also pointed out above. We are happy to present an updated assembly where these large differences have been resolved. On the one hand, this is due to updated dosage estimates of nodes thanks to the added support by newly generated ONT sequencing data. On the other hand, most of the differences could be attributed to a number of contigs remaining unphased after the clustering step. To solve this, we have added a postprocessing step: of each of the remaining contigs, a contig is added to the cluster with highest correlation to the node, if a clear result emerges.*

*Repeating what we have answered to Reviewer 1:*

*We have updated Figure 4 accordingly, showing that the haplotypes of ch07, ch10 and ch12 - which were formerly problematic - now have assimilated in length.*

*We also want to emphasise that contigs that could not be clustered do not need to be completely discarded. They contain valid sequence but not enough unique k-mers to be reliably assigned. We realised that this was not sufficiently clear in our previous figure. Therefore, we have added the information to the plot how many nodes were included in the first clustering step to form the chromosomal cluster, but had to be excluded from the second step of phased clustering due to not being phase-informative (= lacking the unique k-mer information). As can be seen in the figure, this "unphased" portion is higher in chromosomes 4, 8 and 10, while e.g. chromosomes 7 and 11 are rather complete.*

*Another factor contributing to the former haplotype size differences in ch10 and ch12 was the following: As highlighted in our later discussion in the paper, we identified a mix-up during the initial chromosomal clustering, leading to the assignment of a problematic component containing parts of two chromosomes to both haplotypes. This mix-up was attributed to an error in hifiasm, incorrectly joining parts of chromosomes 10 and 12 on the same graph component. Consequently, we confidently excluded these nodes from the clustering analysis to avoid misassignments.*

*Concerning ch02, one haplotype still appears larger than expected. However, we have carefully examined this issue and presented a corresponding plot in the supplementary material to demonstrate that it is unlikely to be a misassembly. The nodes from this haplotype indeed map specifically to ch02 in the reference. We have addressed this finding in the manuscript.*

*Also, for a better assessment of the assembly results, we have added the above-mentioned metrics output by Merqury as well as BUSCO.*

**Second round of review**

**Reviewer 1**

The authors generated new data including 162 Gb ONT long reads and performed additional analysis to address the concerns from the first review. Many of them have been addressed, however, the most important one, i.e., how to evaluate and prove the haplotyping accuracy of the proposed method, has not. This is unsatisfactory, because the ultimate goal of the method is to achieve haplotype-resolved genome assembly. Some specific points are listed below.

1. The response regarding there are longer or shorter haplotype sequences for some chromosomes was inadequate.

For example, the first haplotype chromosome of chr02 is longer, while the newly added Suppl. Fig. 6 & 7 cannot demonstrate which part has been duplicated, either biologically (implying true biological sequences) or technically (implying false redundant sequences from assembly), as compared with the DM reference genome. Meanwhile, according to new Suppl. Fig.7, the ends of chr02_h2 and chr02_h3 seemed to miss sequences, while such regions are usually gene-rich which should be in. Do these missing sequences correlate the duplicated sequences of chr02_h0? A similar pattern is observed on chr12.

It would be informative to run a synteny/structural variation analysis to compare the haplotype chromosomes (and DM) between each other. Such an analysis would allow for identifying which parts of a particular chromosome are duplicated or missing, as compared with other haplotypes. This could also be possible to help phase those "unphased" contigs, which are currently randomly assigned to each haplotype (page 11 line 52-55).

2. It seems the proposed method is only able to phase 89.48% of the genome (page 15 line 38). According to the newly added texts (page 6 line 20-22), there is also a drawback of using offspring of two different cultivars for phasing, where the shared sequences between them cannot be phased.

The superiority and drawback of this method should be compared with other published methods, at least with more discussion on this point.

3. Regarding BUSCO analysis to demonstrate completeness.

This should be considered with the goal of the method to achieve haplotypes. The given value of 96.8% on the full tetraploid genome cannot demonstrate how well the haplotypes were resolved. It would be more informative to present the BUSCO score for each haplotype chromosome.

4. The method is proposed for achieving haplotype-resolved genome assembly. Still, I can hardly agree on that the number of contig clusters is a valid indicator of accuracy in phasing or even linkage grouping.

Regarding "We have also created an extended version of Fig. 4a as a new supplementary figure which showcases for each chromosome the four single haplotypes, resulting in a total of 48 mappings to the reference, for a more informative visualisation.", I agree that the sequence alignment can show the sequences belong to a chromosome, i.e, supporting the first step of getting 12 chromosome-related clusters is effective. However, this does not support haplotyping accuracy.

I find it is awkward to say that the parental genomic data of Altus is not available, so there is no direct way to measure the phasing accuracy in this study. Such an evaluation should have been firstly considered in the experimental design.

**Reviewer 2**

This is the manuscript I have reviewed a while ago. I can see the authors have added more data and analyses to this revised version. The authors also confirmed that they want to propose the new METHOD for broad use. Although they have done much work on the final resulted "Haplotype-resolved assembly of a tetraploid genome", I am still not quite convinced whether the approach has a better performance than other available methods.

it is still hard to determine if the final assembly of the Altus genome is truly of high-quality. Problems remain for several chromosomes as showing in the new Figure 4b. There is also lack of sufficient details from the newly added Supplementary Fig 7.

As indicated by the authors, the main difference between this proposed approach and that of Mao et al. is the usage of selfing population data or crossing offspring. The authors also didn't answer whether the genetic divergence of the other cultivar has effect on the final results.

**Authors Response**

**Point-by-point responses to the reviewers’ comments:**

Reviewer #1:

The authors generated new data including 162 Gb ONT long reads and performed additional analysis to address the concerns from the first review. Many of them have been addressed, however, the most important one, i.e., how to evaluate and prove the haplotyping accuracy of the proposed method, has not. This is unsatisfactory, because the ultimate goal of the method is to achieve haplotype-resolved genome assembly. Some specific points are listed below.

*Response: Thank you for these comments. We agree that additional support for the haplotype consistency is valuable. As we describe in detail below, we have now produced additional Pore-C data to address this.*

1. The response regarding there are longer or shorter haplotype sequences for some chromosomes was inadequate.

For example, the first haplotype chromosome of chr02 is longer, while the newly added Suppl. Fig. 6 & 7 cannot demonstrate which part has been duplicated, either biologically (implying true biological sequences) or technically (implying false redundant sequences from assembly), as compared with the DM reference genome. Meanwhile, according to new Suppl. Fig.7, the ends of chr02_h2 and chr02_h3 seemed to miss sequences, while such regions are usually gene-rich which should be in. Do these missing sequences correlate the duplicated sequences of chr02_h0? A similar pattern is observed on chr12.

It would be informative to run a synteny/structural variation analysis to compare the haplotype chromosomes (and DM) between each other. Such an analysis would allow for identifying which parts of a particular chromosome are duplicated or missing, as compared with other haplotypes.

*Response: To analyse structural differences between the chromosomes of our assembly, we have computed synteny in two different ways, using both syntenyPlotteR and SyRI. To prepare the input these methods expect, we ran RagTag to our haplotigs to compute scaffolds. Then, we computed pairwise alignments between the scaffolded haplotypes (h1 to h0, h2 to h1, h3 to h2, as well as h0 to DMv6.1) using minimap2. The resulting synteny data has been input to syntenyPlotteR, resulting in the new Figure 5 (also pasted below). This synteny plot presents an overview of the structural differences between the chromosomal haplotypes, including the reference. The results highlight the (expected) structural diversity between haplotypes while the absence of interchromosomal rearrangements supports the robustness of the chromosomal clustering since there are no false translocations in between different chromosomes.*

*Fig. 5: Synteny analysis. High-level overview of structural differences between chromosomal haplotypes.Top row: DMv6.1, rows 1-4: Haplotypes 0, 1, 2 and 3.*

*For a more detailed overview, we have added synteny plots created with SyRI for each chromosome separately. To do so, we again use the haplotype scaffolds created with RagTag. We have included the resulting 12 plots in the Supplementary Information (Suppl. Fig 17-28).*

*Similarly, we have computed synteny of the scaffolded haplotypes to the reference DMv6.1 and added these plots to the Supplement as well (Suppl. Fig 11-16). For the more complicated chromosomes like chr02 (Supp. Fig. 11/18, pasted below), it is difficult to assess what leads to one haplotype containing more sequence than the others, as there are no large duplications detected. The comparison of the two shorter haplotypes to the chromosomes shows that indeed there is some sequence missing at the beginning of the chromosome. However, it does not seem that a duplicated sequence of the one haplotype corresponds to this sequence. For comparison, we have pasted the corresponding figure from Sun et al. below as well. While this is a different cultivar, large scale rearrangements, including inversions and losses, on chr02 were observed in this study as well.*

*From: Suppl. Fig. 11: Synteny analysis. Comparison between all haplotypes of chr01 (above) (note: not pictured here in the response) and chr02 (below) and the reference sequence DMv6.1.*

This could also be possible to help phase those "unphased" contigs, which are currently randomly assigned to each haplotype (page 11 line 52-55).

*Response: We would like to clarify that there is no step in our approach in which any contig is assigned ‘randomly’ to one haplotype cluster, and this also refers to the 'unphased' contigs which could not be added to one haplotype cluster before. This comment probably refers to the post-processing step we introduced (page 29, lines 16-20). Instead, contigs are only added to a haplotype cluster if there is enough evidence in terms of high correlation. In this sense our method in conservative by only assigning contigs to a haplotype if enough evidence is available.*

2. It seems the proposed method is only able to phase 89.48% of the genome (page 15 line 38). According to the newly added texts (page 6 line 20-22), there is also a drawback of using offspring of two different cultivars for phasing, where the shared sequences between them cannot be phased. The superiority and drawback of this method should be compared with other published methods, at least with more discussion on this point.

*Response: It is important to point out the different research setting that our method can be applied in, which is particularly within the domain of applied research. In the context of such applied research environments, research goals often involve breeding experiments, aimed at enhancing traits, for instance. There, the practice of crossbreeding distinct cultivars is the norm, and we here show that our method yields a reliable genome assembly tailored to such settings. While it is indeed challenging to work with offspring data from such crossbreeding experiments, overcoming these challenges was a key motivation for our study. That is, our approach is applicable to settings where the crosses are already available and can be leveraged to create the phased assemblies. This avoids the need to create either pollen sequencing or self-crosses specifically for genome assembly. Therefore, our motivation was not so much to demonstrate that the assemblies are superior, but to show that assemblies of comparable quality can be produced in the more challenging scenario of crosses of distinct cultivars.*

*This setting indeed comes with the drawback of having to exclude some sequence that cannot be reliably used for phasing. Still, we would like to clarify that shared sequence between Altus and Colomba can be phased in principle by our method. We do exclude kmers that also appear in the Colomba genome in the step that chooses unique k-mers as markers for nodes. This is inherent to our clustering approach and necessary so that only the two Altus haplotypes are detected in the offspring data. However, this step only excludes (the often short) nodes which do not carry any Altus-specific k-mers at all. Other nodes which may contain shared sequence between the two cultivars, but also at least some Altusspecific sequence within, allowing us to identify the node, can be phased. Also, see our response to Comment 2 of Reviewer 2, where we have analysed the effect that shared sequence might have on the phasing outcome and found that indeed the ‘divergence’ of a node (as measured by the fraction of Altus-specific k-mers per node, i.e. k-mers not present in the Colomba genome) has an influence on whether or not a node can be phased.*

*We have now added further clarification on this to the Conclusion and Discussion section.*

3. Regarding BUSCO analysis to demonstrate completeness.

This should be considered with the goal of the method to achieve haplotypes. The given value of 96.8% on the full tetraploid genome cannot demonstrate how well the haplotypes were resolved. It would be more informative to present the BUSCO score for each haplotype chromosome.

*Response: The BUSCO scores per haplotype are as follows: h0: 83.1%, h1: 84%, h2: 81.7%, h3:81.7%. Also, the BUSCO score for the initial hifiasm assembly is 99.0% (97.8% duplicated BUSCOS).*

4. The method is proposed for achieving haplotype-resolved genome assembly. Still, I can hardly agree on that the number of contig clusters is a valid indicator of accuracy in phasing or even linkage grouping.

Regarding "We have also created an extended version of Fig. 4a as a new supplementary figure which showcases for each chromosome the four single haplotypes, resulting in a total of 48 mappings to the reference, for a more informative visualisation.", I agree that the sequence alignment can show the sequences belong to a chromosome, i.e, supporting the first step of getting 12 chromosome-related clusters is effective. However, this does not support haplotyping accuracy.

I find it is awkward to say that the parental genomic data of Altus is not available, so there is no direct way to measure the phasing accuracy in this study. Such an evaluation should have been firstly considered in the experimental design.

*Response: We thank the reviewer for bringing up the point of phasing correctness again. As we pointed out to Comment 2 above, our initial motivation was to devise a method that can work in a common breeding setting where offspring panels are routinely created for trait mapping.*

*To evaluate the phasing correctness, we have now generated ~77 Gb of Pore-C data of Altus. We mapped all the Pore-C reads to the assembly graph nodes (using minimap2) and filtered for MAPQ=50. Next, we detected node pairs that are connected via at least one shared Pore-C read along with their 'coverage', i.e. the number of Pore-C reads covering the node pair, to evaluate our phasing results in two ways:*

*Node pairs which were clustered to the same haplotype (cis pairs) should ideally be supported by Pore-C reads (true positive). On the other hand, node pairs belonging to different haplotypes (trans pairs) should have no Pore-C support (true negative). Parameters that affect the ability of Pore-C to assess a node pair in this way include the distance between both nodes in the graph, the node length and the Pore-C ‘coverage’.*

*To quantify this, we look at pairs of nodes that are longer than 1Mb and apply different distance cutoffs of 3Mb, 5Mb, 7Mb and 10Mb. For each distance, we apply Pore-C coverage cutoffs between 0 and 70 (in steps of 1) as well as some higher cutoffs ranging from 100 to 1200 and compute the corresponding true positive and false positive rates. The corresponding ROC curves are depicted in Suppl. Fig. 29 (see below). The AUC for the different curves are: 0.96, 0.94, 0.93 and 0.93 for the distances of 3Mb, 5Mb, 7Mb and 10Mb, respectively, indicating that the Pore-C data can indeed be used to support the phasing correctness.*

*For a parameter setting consisting of a distance cutoff of 5Mb and a Pore-C coverage cutoff of 30, for instance, we achieve a true positive rate of 95.22% and a true negative rate of 92.56%. In total, 251 cis and 309 trans node pairs fulfil this condition. For node pairs that are further apart (max. 10Mb), which includes a higher number of pairs (502 cis, 706 trans), we are still able to achieve a sensitivity and specificity > 0.9, even though the Pore-C signal expectedly declines with increasing distance.*

*In summary, we see that the newly generated Pore-C data provides a valuable orthogonal datatype to validate the assembled haplotypes, and is concordant to our phasing results.*

*Suppl. Fig 29: ROC curves for four different distance cutoffs. To compute the data points, all Pore-C coverage cutoffs between 0 and 70 (in steps of 1) were tested, as well as some higher cutoffs (ranging between 100 and 1200). The AUC for the different curves are: 0.96, 0.94, 0.93 and 0.93 for the distances of 3Mb, 5Mb, 7Mb and 10Mb, respectively.*

Reviewer #2:

This is the manuscript I have reviewed a while ago. I can see the authors have added more data and analyses to this revised version. The authors also confirmed that they want to propose the new METHOD for broad use. Although they have done much work on the final resulted "Haplotype-resolved assembly of a tetraploid genome", I am still not quite convinced whether the approach has a better performance than other available methods.

it is still hard to determine if the final assembly of the Altus genome is truly of high-quality. Problems remain for several chromosomes as showing in the new Figure 4b. There is also lack of sufficient details from the newly added Supplementary Fig 7.

As indicated by the authors, the main difference between this proposed approach and that of Mao et al. is the usage of selfing population data or crossing offspring.

*Response: To our knowledge, this is the only method that works with offspring of two distinct cultivars, so that it cannot be directly compared to the other published methods that use either singlecell data or offspring from a selfing population. This makes our method the only one which is tailored to applied research, in which crossing two different cultivars (often with the aim of breeding scenarios for enhanced traits) is the standard (also see our response to Comment 2 of Reviewer 1). Additionally, many plants cannot be inbred at all. Potato itself is an outbreeding crop. It can be inbred to a certain degree, but still crosses of different lines are common in breeding research. Therefore this application scenario is a realistic one. We now provide a more nuanced discussion in the manuscript where we highlight the ability to work with offspring from different cultivars both as a practical advantage but also the drawback (slightly lower phasing completeness) that comes with this method in contrast to others.*

*For a more thorough evaluation on the final assemblies, we have performed synteny analysis to visualise structural differences between the final haplotypes themselves, as well as the haplotypes and the DMv6.1 reference sequence, resulting in Fig. 5 and Supplemental Figures 11-28. In general, these synteny plots show high synteny among the chromosomes and the reference and visualise remaining structural differences for the more complicated chromosomes. Still, this is another indicator for the robustness of the chromosomal clustering, as there are no translocations between the distinct chromosomes.*

*Further, to evaluate, we have generated Pore-C data for Altus and used mappings of these reads to the contigs in order to validate node pairs which have been assembled on the same haplotype, see our response to Comment 1 of Reviewer 1 above. In brief, we have identified rates of true positives and true negatives (= pairs on the same haplotype which have Pore-C support, or pairs on different haplotypes which have no Pore-C support, respectively) for different cut-offs of Pore-C coverage and distance between the nodes in the genome. We were able to show that 95.22% of node pairs on the same haplotype were sufficiently supported by Pore-C reads, while 92.56% of node pairs on different haplotypes correctly showed no Pore-C support. Given the noisy nature of Pore-C data, we consider this to be very good validation rates supporting the phasing correctness of our assemblies.*

The authors also didn't answer whether the genetic divergence of the other cultivar has effect on the final results.

*Response: We thank the reviewer for raising this point again. To get an estimate of the ‘divergence’ of a node, we computed the k-mers that are shared between the Altus and the Colomba genome. As a proxy for ‘divergence’, we define the specific k-mer fraction (SKF) by dividing the number of Altus-specific k-mers (i.e., k-mers that are not present in Colomba) by the total number of k-mers of a node. This SKF value is 0 when there are no k-mers specific for Altus in a node, i.e. all k-mers are shared with Colomba, and it is 1 when all k-mers are uniquely present in Altus.*

*To estimate the effect of SKF of a node on the phasing outcome, we performed a regression using the length, coverage/dosage and SKF as predictors (independent variables) to predict the probability of being phased (response). The coefficient estimates for the multiple logistic regression model are given as follows:*

*length 5.450e-06, divergence rate 0.01861, coverage 0.01247 (the p-values associated with all variables are < 0.001), indicating that each of these variables is significantly associated with the probability of being phased, which is in line with our expectation. So indeed the divergence (as measured by SKF) has an influence on whether or not a node can be phased, even when correcting for the two confounding factors of node length and coverage/dosage.*

*For example, for a node length of 200kbp and coverage of 32 (associated with dosage=1), we can predict that if such a node has a low SKF of 0.2, it has a probability of 0.33 to be phased, while with a high fraction of specific k-mers of 0.8, this probability increases to 0.6.*

*We observe the following distribution of the fraction of Altus-specific k-mers (SKF):*

**Third round of review**

**Reviewer 1**

The authors addressed most of my last concerns, and they have generated ~77 Gb Pore-C data to evaluate the phasing accuracy. While this could show the power of the method in long (but still local) haplotype phasing, it also resulted in a few new questions (given below). In addition, the completeness is indeed not very good as shown by Figure 5 (where the triangle gaps between two compared haplotypes might indicate missing sequences in the shorter one) and BUSCO scores per haplotype (82-84%), where the authors did give some potential reasons on this in the discussion part.

Page 16:

1. Texts need to be polished substantially.

2. Why not using Pore-C to perform de novo scaffolding (to avoid reference bias)?

Page 18:

1. Texts need to be polished substantially.

2. It would be interesting to show additional statistics on Pore-C data, including the length distribution of all Pore-C reads, as well as the distribution of the number of Pore-C read pairs linking two nodes.

3. Since the assembly graph was used for Pore-C reads mapping (lines 1-2), contig nodes with dosage >=2 will join multiple haplotypes with Pore-C read pairs, is this correct? How to handle this?

4. It would be interesting to show the coverage of Pore-C reads along the scaffolded chromosomes. How uniform was the coverage? Were there any regions that could not be covered by any Pore-C reads?

**Reviewer 2**

The authors have done additional analyses to address several questions. In general, I think the authors have done their best to answer these questions. I have no further comments.

**Authors Response**

**Point-by-point responses to the reviewers’ comments:**

Reviewer #1:

The authors addressed most of my last concerns, and they have generated ~77 Gb Pore-C data to evaluate the phasing accuracy. While this could show the power of the method in long (but still local) haplotype phasing, it also resulted in a few new questions (given below). In addition, the completeness is indeed not very good as shown by Figure 5 (where the triangle gaps between two compared haplotypes might indicate missing sequences in the shorter one) and BUSCO scores per haplotype (82-84%), where the authors did give some potential reasons on this in the discussion part.

Page 16:

1. Texts need to be polished substantially.

*Response: We have now revised the text.*

2. Why not using Pore-C to perform de novo scaffolding (to avoid reference bias)?

*Response: Thank you for the suggestion. While it is true that performing scaffolding using a reference introduces some bias, on the other hand, it is also a reliable option, while de novo scaffolding using Pore-C might integrate new potential errors, which we wanted to mitigate. Additionally, our Pore-C reads are relatively short, which may impact their effectiveness in significantly improving the N50 of scaffolded contigs. In our approach, we therefore prioritized a more conservative strategy by opting for an existing reference. Nevertheless, exploring the possibility of the Pore-C reads for scaffolding might be something to integrate in future research.*

Page 18:

1. Texts need to be polished substantially.

*Response: We gone through the text and have revised it to improve clarity.*

2. It would be interesting to show additional statistics on Pore-C data, including the length distribution of all Pore-C reads, as well as the distribution of the number of Pore-C read pairs linking two nodes.

*Response: See below the distribution of the number of Pore-C contacts:*

*with an average of 6.17 and a maximum value of 3052. This refers to the same dataset that was used for the quality control in the manuscript, i.e. mapping qualities are 50 or higher.*

*The length distribution of all Pore-C reads is given as follows:*

*with a maximum length of 528,270 bases and a mean length of 780 bases.*

3. Since the assembly graph was used for Pore-C reads mapping (lines 1-2), contig nodes with dosage >=2 will join multiple haplotypes with Pore-C read pairs, is this correct? How to handle this?

*Response: Nodes with dosage >= 2 may indeed be part of multiple haplotypes. In this case, for each of the contained haplotypes, the Pore-C-based verification takes place just as with dosage 1 nodes: Suppose a node n with dosage 2 is part of two haplotype clusters; cluster 1 containing a node n’ and cluster 2 containing a node n”. Then we can verify both pairings using the number of Pore-C read pairs covering (n, n’) as well as (n, n”). There is no problem associated with higher dosage nodes since we do not take the Pore-C ‘coverage’ per node into account, but per* ***node pair****. Thus, for dosage d of a candidate node, d node pairs may be verified individually by the Pore-C data.*

4. It would be interesting to show the coverage of Pore-C reads along the scaffolded chromosomes. How uniform was the coverage? Were there any regions that could not be covered by any Pore-C reads?

*Response: We computed the coverage of Pore-C reads using samtools. The coverage among all scaffolded chromosomes was very high (see table below), with very few exceptions, the majority of chromosomes is covered by more than 95%. There were no larger regions that went completely uncovered by any reads.*

*The percentage of covered bases per haplotype is given as (in the order of hapotype 0, 1, 2, 3):*

*chr01	91.53%, 98.1%, 91.76%, 92.22%*

*chr02	98.39%, 98.2%, 97.39%, 98.84%*

*chr03	88.16%, 84.41%, 90.22%, 97.78%*

*chr04	76.98%, 86.89%, 87.17%, 83.54%*

*chr05	95.76%, 97.84%, 98.52%, 98.31%*

*chr06	96.92%, 97.9%, 98.05%, 98.62%*

*chr07	92.86%, 95.88%, 96.26%, 95.38%*

*chr08	94.35%, 97.33%, 96%, 98.48%*

*chr09	93.21%, 95.27%, 95.84%, 94.95%*

*chr10	70.82%, 70.68%, 74.03%, 77.5%*

*chr11	98.64%, 95.56%, 93.93%, 95.22%*

*chr12	96.02%, 97.6%, 97.52%, 98.62%*
